# Ribosomal S6K1 in POMC and AgRP Neurons Regulates Glucose Homeostasis but Not Feeding Behavior in Mice

**DOI:** 10.1016/j.celrep.2015.03.029

**Published:** 2015-04-09

**Authors:** Mark A. Smith, Loukia Katsouri, Elaine E. Irvine, Mohammed K. Hankir, Silvia M.A. Pedroni, Peter J. Voshol, Matthew W. Gordon, Agharul I. Choudhury, Angela Woods, Antonio Vidal-Puig, David Carling, Dominic J. Withers

**Affiliations:** 1 Metabolic Signalling Group, Medical Research Council Clinical Sciences Centre, Imperial College London, Hammersmith Campus, London W12 0NN, UK; 2 University of Cambridge, Metabolic Research Laboratories, MRC Metabolic Diseases Unit, Wellcome Trust-MRC Institute of Metabolic Science, Level 4, Box 289, Addenbrooke’s Hospital, Cambridge CB2 0QQ, UK; 3 Cellular Stress Group, Medical Research Council Clinical Sciences Centre, Imperial College London, Hammersmith Campus, London W12 0NN, UK

## Abstract

Hypothalamic ribosomal S6K1 has been suggested as a point of convergence for hormonal and nutrient signals in the regulation of feeding behavior, bodyweight, and glucose metabolism. However, the long-term effects of manipulating hypothalamic S6K1 signaling on energy homeostasis and the cellular mechanisms underlying these roles are unclear. We therefore inactivated S6K1 in pro-opiomelanocortin (POMC) and agouti-related protein (AgRP) neurons, key regulators of energy homeostasis, but in contrast to the current view, we found no evidence that S6K1 regulates food intake and bodyweight. In contrast, S6K1 signaling in POMC neurons regulated hepatic glucose production and peripheral lipid metabolism and modulated neuronal excitability. S6K1 signaling in AgRP neurons regulated skeletal muscle insulin sensitivity and was required for glucose sensing by these neurons. Our findings suggest that S6K1 signaling is not a general integrator of energy homeostasis in the mediobasal hypothalamus but has distinct roles in the regulation of glucose homeostasis by POMC and AgRP neurons.

## Highlights

• Mice lacking S6K1 in POMC and AgRP neurons have impaired glucose homeostasis
• S6K1-deficient POMC and AgRP neurons have altered excitability
• S6K1 in POMC and AgRP neurons does not regulate food intake and bodyweight

## Graphical Abstract

**Figure:**
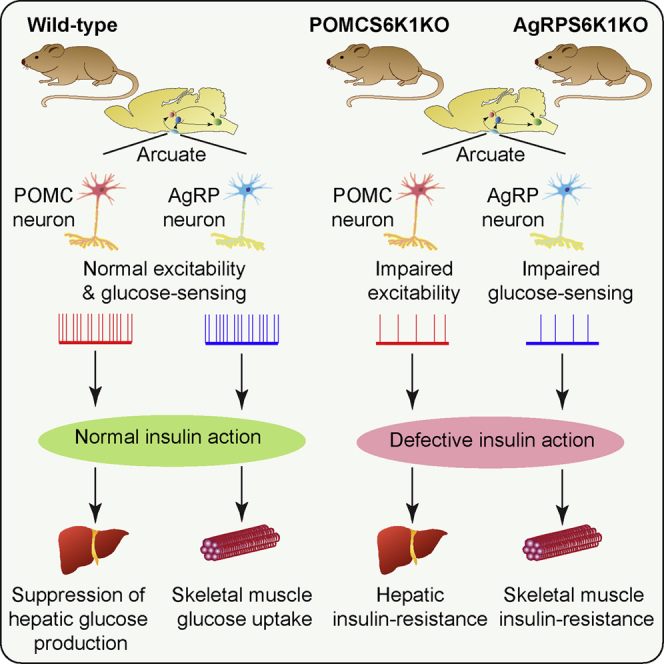

## Teaser

POMC and AgRP neurons regulate systemic energy homeostasis. Smith et al. demonstrate that S6K1 signaling in these cells regulates neuronal excitability and peripheral glucose homeostasis in mice. However, POMC and AgRP S6K1 signaling does not play a major role in feeding behavior and the control of bodyweight.

## Introduction

Obesity and its associated diseases such as type 2 diabetes and cancer are major causes of morbidity and premature mortality (Kopelman, 2000). The CNS orchestrates food intake, nutrient storage, energy expenditure, and related behaviors (Morton et al., 2014; Williams and Elmquist, 2012). Key neuronal populations have been identified that respond to both hormonal and nutrient signals that encode information about the internal metabolic milieu and environmental factors such as diet and stress (Williams and Elmquist, 2012). For example, anorexigenic pro-opiomelanocortin (POMC) and orexigenic agouti-related peptide (AgRP)-expressing neurons in the hypothalamic arcuate nucleus coordinately regulate food intake and energy expenditure as well as peripheral tissue glucose homeostasis and energy partitioning (Varela and Horvath, 2012).

A significant body of work has focused on identification of common intracellular signaling pathways that respond to a range of metabolic signals and may couple hormone and nutrient sensing by neurons to the regulation of organismal metabolism. One such pathway is the mechanistic target of rapamycin (mTOR) signaling network (André and Cota, 2012; Blouet and Schwartz, 2010; Haissaguerre et al., 2014). mTOR exists in two distinct multimolecular complexes, mTORC1 and mTORC2, which are thought to subserve distinct cellular roles (Laplante and Sabatini, 2012). mTORC1 is responsive to a range of factors, including growth factors, amino acids, energy status, and oxygen and has been implicated in the regulation of protein synthesis, lipogenesis, nucleic acid synthesis, and other processes (Laplante and Sabatini, 2012). mTORC1 regulates, through phosphorylation, a number of downstream effector proteins, the best characterized of which are p70 ribosomal S6 protein kinase-1 (S6K1) and eukaryotic translation initiation factor-4E (eIF4E)-binding protein-1 (4E-BP1) (Laplante and Sabatini, 2012). S6K1 has been implicated in ribosomal biogenesis and translational regulation as well as the control of cell size, gene transcription, and the feedback regulation of insulin signaling (Magnuson et al., 2012).

Hypothalamic mTORC1 signaling and S6K1 in particular have been linked to the regulation of energy homeostasis (Blouet and Schwartz, 2010; Haissaguerre et al., 2014). S6K1 is expressed in the hypothalamus, including in POMC and AgRP neurons where it is sensitive to nutrient status (Cota et al., 2006). Hormonal regulators of feeding behavior and bodyweight such as leptin, insulin, and ghrelin modulate hypothalamic S6K1 signaling (Zhang et al., 2011). Pharmacological inhibition of mTORC1 in the hypothalamus using rapamycin blocks the anorectic effect of leptin, leucine, and ghrelin (Cota et al., 2006; Martins et al., 2012), and mice globally lacking S6K1 are resistant to the anorectic effects of leptin (Cota et al., 2008). Adenovirally mediated acute overexpression of a constitutively active S6K1 in the mediobasal hypothalamus (MBH) of rats suppresses food intake, lowers weight gain, and improves insulin sensitivity (Blouet et al., 2008), while dominant-negative S6K1 increases food intake and weight gain, suggesting bidirectional regulation of energy homeostasis by S6K1 (Blouet et al., 2008). Mediobasal hypothalamic S6K1 may regulate peripheral glucose metabolism although, in contrast to the findings described above, constitutive activation of S6K1 results in hepatic insulin resistance (Ono et al., 2008). These observations suggest that mTORC1 and S6K1 in particular may play a key integrative role in hypothalamic nutrient and hormonal sensing and the regulation of energy homeostasis.

Despite these findings, a number of issues remain unclear with respect to the role of S6K1 in the hypothalamus. For example, the use of acute non-targeted viral systems to manipulate S6K1 signaling has not permitted long-term studies on energy balance and glucose homeostasis or revealed any specific contribution of POMC and AgRP neurons. The effects of hypothalamic S6K1 on peripheral glucose metabolism also require clarification (Blouet et al., 2008; Ono et al., 2008). Finally, there is little information on how altered hypothalamic S6K1 signaling might mechanistically regulate neuronal function and energy homeostasis. We therefore generated mice lacking S6K1 (*Rps6kb1*) specifically in either POMC or AgRP neurons. In contrast to published studies, we found a minimal role for S6K1 in the long-term regulation of food intake and bodyweight. S6K1 in POMC and AgRP neurons, however, played a key role in neuronal excitability and the regulation of peripheral glucose homeostasis.

## Results

### Mice with Deletion of S6K1 in POMC and AgRP Neurons

**Figure 1:**
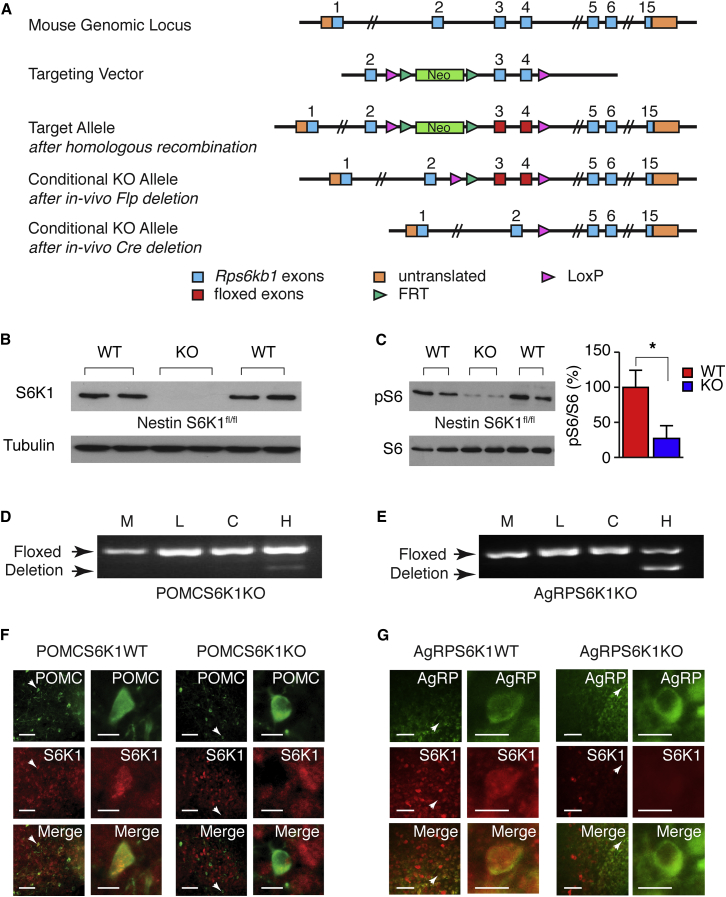
Generation of Conditional *Rps6kb1* Floxed Mice (A) Schematic diagram of the targeting approach for *Rps6kb1* before and after homologous recombination into the mouse genomic locus. The neomycin (Neo) resistance gene was removed by breeding with *Flpe* mice. (B) Representative western blot analysis of S6K1 from brain lysates of nestin-cre S6K1^fl/fl^ WT and KO mice. (C) Western blots of phosphorylated S6 (pS6, top) and total S6 (bottom) in brain lysates from nestin-cre S6K1^fl/fl^ WT and KO mice. Bar chart of the ratio between pS6 and total S6 is show on the right. n = 4–5 mice per genotype, mean ± SEM, ^∗^p < 0.05. (D and E) Representative PCR analysis for the floxed allele (top) and recombination (deletion) event (bottom) in POMCS6K1KO (D) and AgRPS6K1KO (E) mice. M, skeletal muscle; L, liver; C, cerebral cortex; H, hypothalamus. (F and G) Fluorescent (green) arcuate POMC (F) and AgRP (G) neurons in WT (left two panels) and KO (right two panels) mice are shown adjacently at low and high magnifications. Representative S6K1 labeled neurons (red) co-localize with POMC and AgRP neurons in WT but not *Rps6kb1*-deleted cells. Scale bars represent 50 and 10 μm for low- and high-magnification images, respectively.

Mice were generated on a C57Bl/6 background with exons 3 and 4 of *Rps6kb1* flanked by loxP sites (Figure 1A). When bred to homozygosity, *Rps6kb1* floxed (S6K1^fl/fl^) mice were phenotypically indistinguishable from WT animals and displayed normal *Rps6kb1* expression in the absence of cre-recombinase (data not shown). In mice with either germ-line deletion or with global neuronal deletion (using either nestin-cre or synapsin-cre transgenic mice), western blotting confirmed complete loss of S6K1 in all tissues or specifically in the CNS, respectively (Figure 1B; data not shown). Phosphorylated S6 was significantly reduced in whole-brain lysates from nestin-cre S6K1^fl/fl^ mice (Figure 1C). S6K1^fl/fl^ mice were subsequently bred with mice expressing cre-recombinase driven by *Pomc* or *Agrp* promoters (POMCS6K1 or AgRPS6K1, respectively), and the recombination event was restricted to the hypothalamus in both knockout (KO) lines (Figures 1D and 1E). S6K1 immunoreactivity was co-localized with WT fluorescently labeled POMC and AgRP arcuate neurons, but expression was absent in *Rps6kb1*-deleted neurons (Figures 1F and 1G). POMC cre-recombinase mice also drive deletion in pituitary corticotrophs, but corticosterone levels were equivalent between WT and POMCS6K1-KO mice (WT, 41.3 ± 7.7 versus KO, 45.3 ± 7.7 ng/ml, n = 4–6).

### Assessment of Feeding and Bodyweight Phenotypes in POMCS6K1KO and AgRPS6K1KO Mice

**Figure 2:**
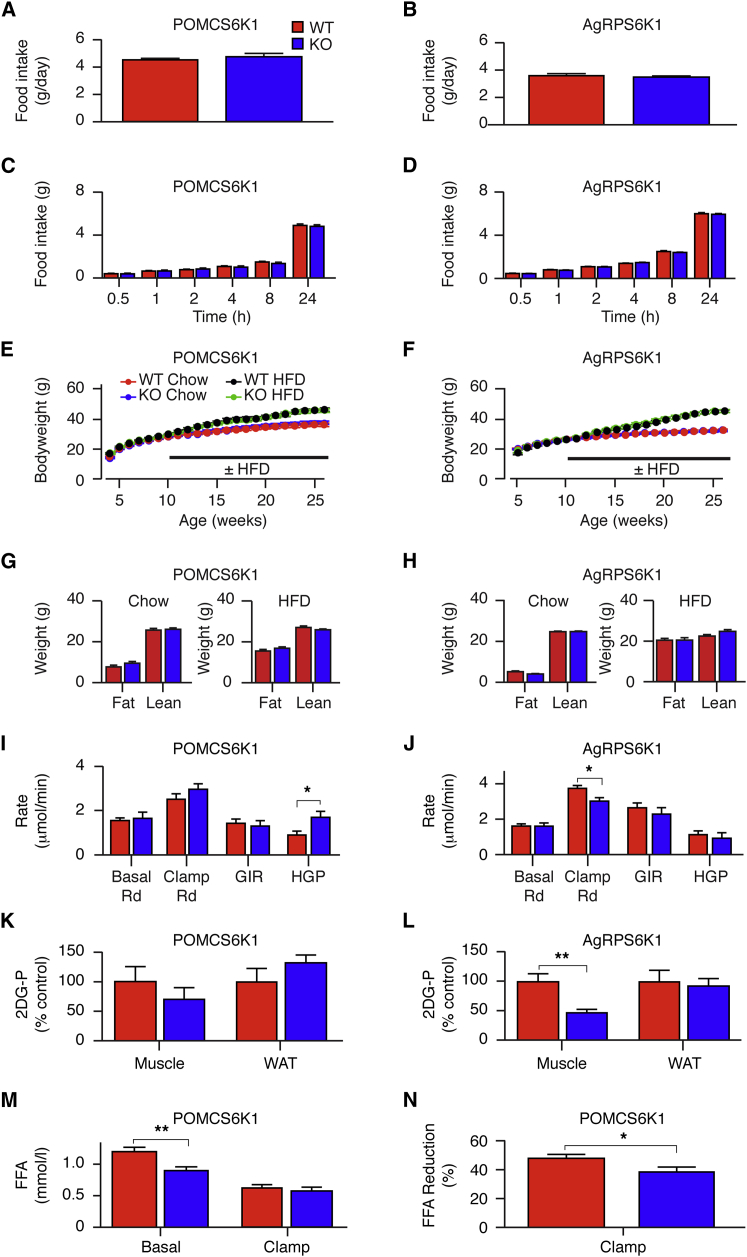
POMC and AgRP S6K1 Regulate Peripheral Glucose Homeostasis but Not Feeding Behavior and Bodyweight (A and B) Ad libitum food intake in male WT (red bars) and KO (blue bars) POMCS6K1 (A) and AgRPS6K1 (B) mice on normal chow. POMCS6K1, n = 20–23, and AgRPS6K1, n = 7–10 mice per genotype. Mean ± SEM. (C and D) Cumulative food intake from WT (red) and KO (blue) male POMCS6K1 (C) and AgRPS6K1 (D) mice following an overnight fast. POMCS6K1, n = 20–23, and AgRPS6K1, n = 7–10, mice per genotype. Mean ± SEM. (E and F) Bodyweight curves for male POMCS6K1 (E) and AgRPS6K1 (F) mutant mice on normal chow (n = 18–22 and n = 23–33 mice per genotype, respectively) or on a HFD (n = 19–21 and n = 19–26 mice per genotype, respectively). WT mice are shown by red circles on chow and black circles on HFD, whereas KO mice on chow or HFD are shown in blue or green circles, respectively. Mean ± SEM. (G and H) EchoMRI analysis of WT (red) and KO (blue) mice shown in (E) and (F). Fat and lean mass at 34 weeks old in POMCS6K1 (G) and AgRPS6K1 (H) mice on normal chow (left) or HFD (right). Mean ± SEM. (I and J) Glucose disposal (Rd), GIR and HGP during basal and hyperinsulinemic-euglycemic clamp conditions from WT (red) and KO (blue) POMCS6K1 (I) and AgRPS6K1 (J) mice. n = 8–9 mice per genotype, mean ± SEM, ^∗^p < 0.05. (K and L) Skeletal muscle and white adipose tissue (WAT) uptake of ^14^C-2-deoxyglucose-phosphate (2-DG-P) from WT (red) and KO (blue) POMCS6K1 (K) and AgRPS6K1 (L) mice shown in (I) and (J). Mean ± SEM, ^∗∗^p < 0.001. (M) Serum FFA concentration before and the during hyperinsulinemic-euglycemic clamp in WT (red) and KO (blue) POMCS6K1 mice shown in (I). Mean ± SEM, ^∗∗^p < 0.001. (N) Percentage reduction of serum FFA concentration following hyperinsulinemic-euglycemic clamp in WT (red) and KO (blue) POMCS6K1 mice shown in (I). Mean ± SEM, ^∗^p < 0.05.

Food intake, ad libitum and in response to a fast, was unaltered in POMCS6K1KO and AgRPS6K1KO mice on chow (Figures 2A–2D), and average meal size, meal number, or feeding pattern over a 24-hr period were not different (Figures S1A–S1D). Both mutant lines responded normally to leptin and AgRPS6K1KO mice displayed an unaltered response to ghrelin (Figures S1E–S1G). Central neuropeptide Y (NPY, 1 μg) evoked rapid feeding, but administration of leucine (2.2 μg) to the same animals did not suppress feeding in both WT and POMCS6K1KO mice (Figures S1H and S1I). POMCS6K1KO and AgRPS6K1KO mice responded normally to the effects of the melanocortin agonist MTII (Figures S1J and S1K). As decreased melanocortin receptor signaling can enhance adiposity in the absence of hyperphagia (Nogueiras et al., 2007), we examined long-term bodyweight regulation on chow and high-fat diet (HFD). Up to 34 weeks of age, there were no differences in bodyweight, fat mass, and serum leptin concentrations between POMCS6K1KO or AgRPS6K1KO mice and their respective controls in either gender (Figures 2E–2H, S1L, and S1M; data not shown). POMCS6K1KO mice had a modest increase in fasted serum leptin levels on a HFD at 34 weeks of age despite their normal adiposity (Figure S1L). Light- or dark-phase energy expenditure, respiratory exchange ratio, and locomotion were unchanged between chow-fed WT and POMCS6K1KO or AgRPS6K1KO mice (Table S1). These data suggest that ablation of *Rps6kb1* signaling in POMC and AgRP neurons does not affect food intake or long-term bodyweight regulation.

### Glucose Homeostasis in POMCS6K1 and AgRPS6K1 Mice

As POMC and AgRP neurons regulate peripheral glucose homeostasis, we studied this in POMCS6K1KO or AgRPS6K1KO mice on chow and HFD. Fed or fasted serum glucose levels, fasted insulin levels (Table S2), and glucose tolerance were not different between control and mutant mice regardless of age (8 and 26 weeks) or diet (data not shown), while POMCS6K1KO mice had a mild impairment in insulin sensitivity on chow (area under the curve: WT, 79.5 ± 2.0 versus KO, 86.7 ± 3.1, n = 20–22, p < 0.05).

Hyperinsulinemic-euglycemic clamp studies were conducted to reveal any potential tissue-specific regulation of glucose homeostasis. Serum glucose concentrations, glucose infusion rate (GIR), and insulin concentrations before and at the end of the clamp were equivalent between male WT and POMCS6K1KO or AgRPS6K1KO mice (data not shown). Under basal conditions, whole-body glucose utilization, equivalent to endogenous hepatic glucose production (HGP), did not differ between WT and POMCS6K1KO or AgRPS6K1KO mice (Figures 2I and 2J). During the hyperinsulinemic clamp, suppression of HGP was impaired in POMCS6K1KO mice compared with controls (Figure 2I). In contrast, in AgRPS6K1KO mice, whole-body glucose utilization during the clamp was reduced when compared with controls (Figure 2J). While there were no differences in glucose uptake between WT and POMCS6K1KO mice (Figure 2K), glucose uptake into skeletal muscle was impaired in AgRPS6K1KO mice (Figure 2L).

In POMCS6K1KO compared with control mice, fasted plasma free fatty acid (FFA) levels were lower but not suppressed by the hyperinsulinemic clamp (Figures 2M and 2N). FFA levels were not different between WT and AgRPS6K1KO mice under basal (WT, 0.87 ± 0.05 versus KO, 1.07 ± 0.05 mmol/l, n = 10–11) and clamp conditions (WT, 0.40 ± 0.05 versus KO, 042 ± 0.03 mmol/l, n = 8). Total triglyceride levels were unaltered in both lines (POMC: WT, 107.7 ± 5.7 versus KO, 101.0 ± 5.5 mg/dl, n = 13; AgRP: WT, 121.4 ± 15.3 versus KO, 124.5 ± 11.4 mg/dl, n = 8–10). Together these findings indicate that loss of *Rps6kb1* in POMC neurons impairs central regulation of HGP and also impacts upon peripheral lipid metabolism, while loss of *Rps6kb1* in AgRP neurons impairs skeletal muscle insulin sensitivity.

### Mechanisms Underlying Metabolic Phenotypes in POMCS6K1KO and AgRPS6K1KO Mice

The liver expression of phosphoenolpyruvate carboxykinase-1 (*Pck1*), glucose-6-phosphatase, and interleukin-6 (a cytokine implicated in the control of HGP) were unaltered in either POMCS6K1KO or AgRPS6K1KO mice (Figures S2A and S2B). Consistent with our finding of reduced FFA levels in POMCS6K1KO mice, we found that adipose tissue mRNA levels of hormone-sensitive lipase-1 were reduced (Figure S2C), whereas those of stearoyl-CoA desaturase-1, fatty-acid synthase, diacylglycerol o-acyltransferase-2, fatty-acid binding protein-4, perilipin-1, and *Pck1* were unaltered in both POMCS6K1KO and AgRPS6K1KO mice (Figures S2C and S2D). To study connections between the melanocortin system and peripheral metabolism, we examined sympathetic nervous system function. Urinary norepinephrine and epinephrine concentrations, core temperature, and brown adipose tissue gene expression were unaltered in both POMCS6K1KO and AgRPS6K1KO mice, suggesting no major alteration in sympathetic function (Figures S2E–S2G; data not shown). Next we examined potential hypothalamic mechanisms that might underlie the changes in peripheral metabolism in POMCS6K1KO and AgRPS6K1KO mice. Hypothalamic *Agrp*, *Npy*, *Pomc*, carboxypeptidase E, and melanocortin-4 receptor mRNA expression was unaltered in POMCS6K1KO and AgRPS6K1KO mice (Figures S2H and S2I). Phosphorylation of signal transducer and activator of transcription-3 (pSTAT3), a major mediator of leptin action, was equivalent between WT and POMCS6K1KO mice (Figures S3A–S3C). S6K1 has also been reported to phosphorylate AMP-dependent protein kinase (AMPK) on serine 485/491 to mediate leptin’s effect on food intake (Dagon et al., 2012). However, we found no differences in hypothalamic AMPK serine 485 phosphorylation after leptin treatment in WT and mice lacking *Rps6kb1* in all neurons (Figure S3D).

In electrophysiology studies, leptin depolarized a sub-population of WT (9 of 16) and *Rps6kb1*-deleted (8 of 15) POMC neurons (Figures S3E, S3F, and S3J). Similar to our previous studies (Al-Qassab et al., 2009; Claret et al., 2007), insulin modestly depolarized a subpopulation of WT (6 of 12 neurons; Figures S3G and S3J) but also 4 of 12 *Rps6kb1*-deleted AgRP neurons studied (Figure S3H). However, insulin also hyperpolarized 2 of 12 *Rps6kb1*-deleted AgRP neurons; thus, the population response to insulin as a whole showed no significant change (Figures S3I and S3J). Although intracerebroventricular (i.c.v.) leucine did not alter food intake, leucine (5 mM) modestly depolarized WT POMC neurons, but equivalent effects were seen in *Rps6kb1*-deleted cells (ΔVm, WT, +3.0 ± 1.1 versus KO, +3.4 ± 1.0 mV, n = 5–6, p < 0.05 from control; Figures S3K and S3L). These findings suggest that S6K1 signaling in POMC neurons is not required for the electrophysiological effects of leptin and leucine and abrogation of S6K1 signaling in AgRP neurons does not impact upon the electrophysiological effects of insulin.

### *Rps6kb1* Deletion Reduces Neuronal Excitability, Alters AgRP Neuron Glucose Sensing and Synaptic Strength and POMC Neuron Size

**Figure 3:**
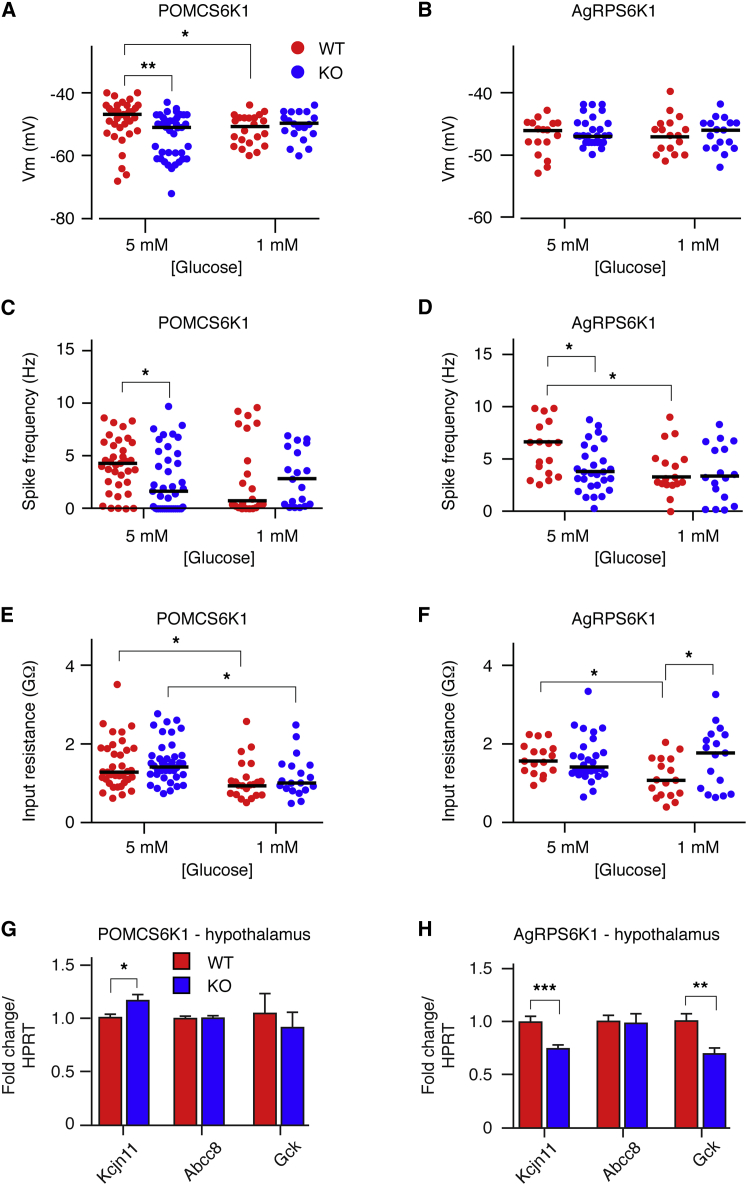
S6K1 Regulates the Excitable Properties of POMC and AgRP Neurons (A–F) Scatter plots of resting membrane potentials (Vm, A and B), spike firing frequencies (C and D), and input resistances (E and F) in POMC (A, C, and E) and AgRP (B, D, and F) neurons in 5 mM (POMC, n = 36–40 and AgRP, n = 17–28 neurons per genotype) or 1 mM (POMC, n = 19–22 and AgRP, n = 17 neurons per genotype) external glucose, as indicated. Solid horizontal bars denote median values. ^∗^p < 0.05, ^∗∗^p < 0.001. WT and KO neurons are shown in red and blue circles, respectively. (G and H) Quantitative PCR for Kir6.2 (*Kcjn11*), SUR1 (*Abcc8*) and glucokinase (*Gck*) from the basomedial hypothalamus of fasted POMCS6K1 (G) and AgRPS6K1 (H) mice. n = 10–12 mice per genotype. Mean ± SEM, ^∗^p < 0.05, ^∗∗^p < 0.001, ^∗∗∗^p < 0.0001. WT and KO neurons are shown in red and blue bars, respectively.

During these studies, it became evident that *Rps6kb1*-deleted POMC and AgRP neurons were less excitable in 5 mM external glucose (Figures 3A–3F), equivalent to fed glucose concentrations in the brain (Routh, 2002). *Rps6kb1*-deleted POMC neurons had a more hyperpolarized resting membrane potential (Vm) and lower spike frequency than control POMC neurons while input resistance was not different (Figures 3A, 3C, and 3E). *Rps6kb1*-deleted AgRP neurons also had a lower resting spike firing frequency, but Vm and input resistance were equivalent to control neurons (Figures 3B, 3D, and 3F). In separate recordings, we examined the excitable properties of POMC and AgRP neurons in 1 mM external glucose, which is similar to fasted glucose concentrations measured in the brain (Routh, 2002). Control POMC neurons had a lower Vm and input resistance when compared with recordings obtained in 5 mM glucose, yet spike frequency was not different (Figures 3A, 3C, and 3E). Input resistance in 1 mM glucose was also lower in *Rps6kb1*-deleted POMC neurons when compared with recordings at the higher glucose concentration but resting Vm and spike firing frequency were equivalent (Figures 3A, 3C, and 3E). In control AgRP neurons, lowering external glucose decreased input resistance and spike frequency when compared with recordings in 5 mM glucose, but Vm was not significantly different (Figures 3B, 3D, and 3F). However, in *Rps6kb1*-deleted AgRP neurons, Vm, spike frequency, and input resistance were equivalent between the two external glucose concentrations (Figures 3B, 3D, and 3F). Therefore, POMC neurons lacking *Rps6kb1* displayed a hyperpolarized resting membrane potential in high glucose while the biophysical properties of AgRP neurons lacking *Rps6kb1* did not alter in different glucose concentrations possibly indicating defective glucose sensing. These defects may be due to altered ATP-sensitive K^+^ (K_ATP_) channel subunit expression, and we found that *Kcjn11* expression was significantly increased in POMCS6K1KO and reduced in AgRPS6K1KO when compared with WT mice; however, *Abcc9* expression was unchanged (Figures 3G and 3H). Hypothalamic expression of glucokinase was reduced in AgRPS6K1KO but unchanged in the POMCS6K1KO mice (Figures 3G and 3H).

**Figure 4:**
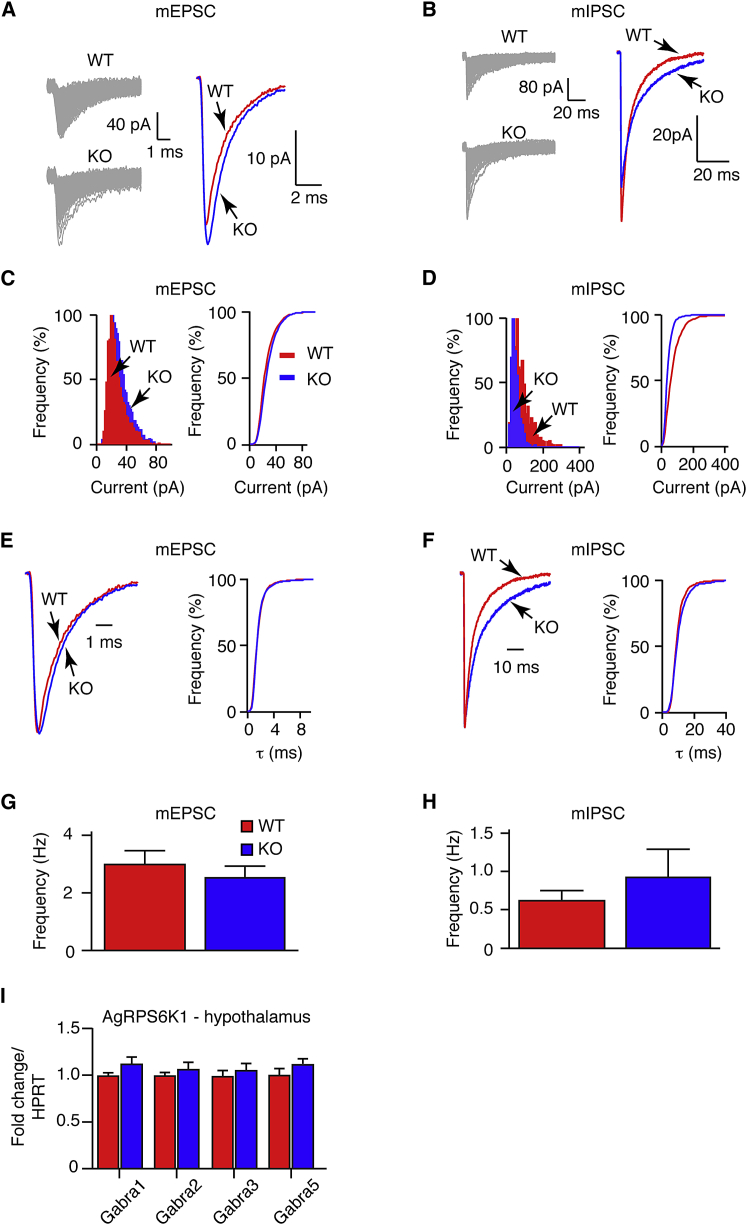
Synaptic Strength in AgRP Neurons Is Altered by *Rps6kb1* Deletion (A and B) Representative overlapping (left) and composite (right) miniature excitatory (A) and inhibitory (B) post-synaptic currents (mEPSC and mIPSC, respectively) in WT and *Rps6kb1*-deleted (KO) AgRP neurons. (C and D) Frequency histograms (left) and cumulative frequency curves (right) are shown for mEPSC (C) and mIPSC (D) amplitudes in WT (red) and KO (blue) AgRP neurons. n = 10–13 neurons per genotype. (E and F) Normalized composite traces (left) with cumulative frequency curves (right) for decay times of mEPSC (E) and mIPSC (F) in WT (red) and KO (blue) AgRP neurons. n = 10–13 neurons per genotype. (G and H) Bar charts of spontaneous mEPSC (G) and mIPSC (H) event frequency in WT (red) and KO (blue) AgRP neurons. n = 10–13 neurons per genotype. Mean ± SEM. (I) Quantitative PCR analysis for GABA_A_ receptor alpha subunits 1, 2, 3, and 5 (*Gabra1*, *Gabra2*, *Gabra3*, *Gabra5*, respectively) in the MBH of fasted WT (red) and KO (blue) AgRPS6K1 mice. n = 10–12 mice per genotype. Mean ± SEM.

Changes in synaptic weight in *Rps6kb1*-deleted AgRP neurons could explain a lower firing frequency without an observable difference in resting Vm. Therefore, we recorded from WT and AgRPS6K1KO neurons and pharmacologically isolated miniature excitatory (mEPSC) and inhibitory (mIPSC) post-synaptic currents in the presence of tetrodotoxin (1 μM; Figures 4A–4H). Mean mEPSC amplitude was slightly higher in *Rps6kb1*-deleted AgRP neurons when compared with WT neurons (WT, −27.2 ± 0.2 versus KO, −29.4 ± 0.2 pA, 4,243–4,505 events from 13 neurons per genotype, p < 0.0001; Figures 4A and 4C). There were no differences in decay time constants or event frequency (Figures 4E and 4G) between WT and KOs. In contrast, mIPSC amplitude was significantly smaller in AgRPS6K1KO neurons when compared with control cells (WT, −81.6 ± 2.1 versus KO, −49.0 ± 0.9 pA, 898–945 events from ten neurons per genotype, p < 0.0001; Figures 4B and 4D). Likewise, mIPSC decay constants were slower in *Rps6kb1*-deleted neurons when compared with WT controls (WT, 9.9 ± 0.1 versus KO, 10.7 ± 0.2 ms, 898–945 events from ten neurons per genotype, p < 0.001; Figure 4F). Spontaneous mIPSC frequency was the same between WT and *Rps6kb1*-deleted AgRP neurons (Figure 4H). Differences in the expression of GABA_A_ receptor alpha subunits may lead to alterations in kinetics, but we found no changes in the expression of GABA_A_ receptor alpha-subunits 1, 2, 3 and 5 in the hypothalamus of AgRPS6K1KO mice (Figure 4I). Together, these data show that chronic deletion of *Rps6kb1* in AgRP neurons alters synaptic strength.

Next, we examined neuronal architecture as S6K1 has been implicated in cell growth. Initial indirect measurement of cell size through cell capacitance measurements did not identify a difference between control and *Rps6kb1*-deleted POMC and AgRP neurons (Figure S4A). However, in immunohistochemistry studies, somatic diameter and area was approximately 5% smaller in *Rps6kb1*-KO when compared with WT POMC neurons (Figures S4B–S4D), while no overt differences in POMC fibers in areas such as the hypothalamic paraventricular nucleus were observed (data not shown).

## Discussion

Hypothalamic S6K1 has been implicated in the regulation of food intake, energy expenditure, and bodyweight (Blouet and Schwartz, 2010; Haissaguerre et al., 2014). In contrast, in our studies specifically ablating *Rps6kb1* in POMC or AgRP neurons, we find that this signaling pathway does not play an indispensable role in regulating these processes. We instead reveal a key role for both POMC and AgRP neuronal S6K1 in peripheral glucose metabolism and show that deletion of *Rps6kb1* alters POMC and AgRP neuron excitability and disrupts glucose sensing in AgRP neurons. Potential explanations for the differences between our findings and previous work include the short-time window but high level of adenovirally mediated gene expression, the lack of cell-selective manipulations, the presence of local hypothalamic inflammation, acute post-operative weight loss, and anorexia complicating the interpretation of feeding and bodyweight data and potential off-target effects of the constructs. Chronic deletion may also lead to compensatory changes such as upregulation of S6K2, but in our preliminary studies deleting both *Rps6kb1* and *Rps6kb2* in POMC neurons, we find no changes in food intake or bodyweight (Figures S4E and S4F). It is also conceivable that the effects of S6K1 on energy balance are due to signaling in other MBH cell populations. Yet, this is unlikely as global neuronal deletion of *Rps6kb1* achieved using nestin-cre mice did not lead to alterations in bodyweight or insulin tolerance (Figures S4G and S4H). In contrast, our broad range of relevant physiological, molecular, and electrophysiological data indicate that S6K1 is not required for the hypothalamic regulation of food intake, bodyweight, and energy expenditure.

Rapamycin blocks the anorexigenic effects of leptin (Cota et al., 2006), but the lack of effect of *Rps6kb1*-deletion on acute leptin regulation of both food intake and POMC neuron excitability and signaling suggests the involvement of an alternative rapamycin-sensitive mechanism. S6K1 also phosphorylates AMPK on serine 485/491, an event that inhibits AMPK activity and is required for leptin’s effect on food intake (Dagon et al., 2012). However, we were unable to detect differences in AMPK serine 485 phosphorylation after leptin treatment between WT and mice lacking *Rps6kb1* in all neurons. Ghrelin action in AgRPS6K1KO mice was also normal. S6K1 has also been suggested to mediate the anorexigenic effects of central administration of leucine (Blouet et al., 2009; Cota et al., 2008), and while we were unable to detect a suppression of food intake, the persistence of leucine’s electrophysiological actions in *Rps6kb1*-deficient POMC neurons suggest that S6K1 is not required for this component of leucine’s actions.

We find that S6K1 signaling in POMC and AgRP neurons regulates glucose homeostasis. Previous studies have given conflicting results, with one suggesting that hypothalamic activation of S6K1 signaling causes hepatic insulin resistance and its inhibition improves hepatic insulin sensitivity (Blouet et al., 2008), while another showed essentially the opposite findings (Ono et al., 2008). We show that deletion of *Rps6kb1* in POMC neurons leads to defective regulation of HGP, while deletion in AgRP neurons causes skeletal muscle insulin resistance. In POMC neurons leptin receptor signaling alone, or in combination with insulin receptor signaling, and phosphatidylinositol-3 kinase signaling have both been shown to regulate HGP (Berglund et al., 2012; Hill et al., 2010; Hill et al., 2008). Insulin-receptor signaling in AgRP neurons likewise regulates HGP (Könner et al., 2007). Melanocortin circuits control both HGP and insulin sensitivity in skeletal muscle and adipose tissue function (Berglund et al., 2014; Nogueiras et al., 2007; Obici et al., 2001; Rossi et al., 2011). Our studies implicate S6K1 signaling in POMC neurons as one of the mechanisms by which this cell type regulates HGP and peripheral lipid metabolism. We also show a role for AgRP neurons in regulating skeletal muscle sensitivity and implicate S6K1 signaling in this process.

Hypothalamic neuronal excitability is modulated by external glucose by inhibition of K_ATP_ channels following the uptake and metabolism of glucose (Levin et al., 2004). Glucose sensing in POMC neurons has been shown to regulate peripheral glucose homeostasis (Parton et al., 2007), and reducing glucose to hypoglycemic conditions hyperpolarizes both POMC and AgRP neurons (Claret et al., 2007). Consistent with the opening of a resting conductance, input resistance in both POMC and AgRP neuronal populations was lower in an external glucose concentration (1 mM) reflecting the fasted state (Routh, 2002) in comparison with recordings obtained in a higher glucose concentration (5 mM). However, low glucose did not alter the biophysics of AgRPS6K1KO neurons, which may indicate a loss of glucose sensing in these cells, which could underpin alterations in peripheral glucose metabolism. A reduction in the expression of a pore-forming subunit of K_ATP_ channels and the enzyme glucokinase, which initiates glucose metabolism may contribute to this loss of glucose sensing. POMCS6K1KO neurons had a lower input resistance when compared with recordings in higher glucose concentrations, suggesting that glucose sensing is intact. However, these neurons had lower resting membrane potential and spike firing frequency than control POMC neurons, which may imply a reduction in basal transmitter release, which could alter peripheral glucose homeostasis. The properties and kinetics of voltage-dependent conductances or the electrogenic properties of the neuron due to changes in anatomy may be modulated by S6K1 to alter resting excitability. Although cell capacitance measurements in *Rps6kb1*-deficient and control POMC neurons did not suggest alterations, in immunohistochemical anatomical assessments, we found a very small reduction in soma size. One caveat with electrophysiological recordings of individual neurons is that this technique can only capture small samples within the wider neuronal population. In the case of POMC and AgRP neurons, which display some functional heterogeneity and do not all respond to the same hormones and nutrients, it is possible that some neurons that have altered responses are undetected.

In summary, our studies define the physiological and cellular roles of S6K1 signaling in POMC and AgRP neurons and give insights into the role of this pathway in the CNS regulation of energy homeostasis. We find no role for hypothalamic S6K1 in the regulation of feeding and bodyweight but demonstrate a key role for this molecule in glucose homeostasis.

## Experimental Procedures

### Mice and Animal Care

The use and genotyping of POMCCre, AgRPCre (both on a C57Bl/6 background), and NestinCre (on a mixed C57Bl/6 × 129sv background) mice have been previously described (Al-Qassab et al., 2009; Choudhury et al., 2005; Claret et al., 2011). Mice with floxed alleles for *Rps6kb1* on a C57Bl/6 background were generated by Taconic Biosciences and crossed with the *Cre*-expressing transgenic mice to generate compound heterozygotes. These mice were intercrossed with *Rps6kb1*^*fl/wt*^ mice to obtain WT (*Cre*^*−/−*^*/Rps6kb1*^*wt/wt*^, *Cre*^*−/−*^*/Rps6kb1*^*fl/fl*^, *Cre*^*+/−*^*/Rps6kb1*^*wt/wt*^), and KO (*Cre*^*+/−*^*/Rps6kb1*^*fl/fl*^) mice for each line. To generate mice lacking floxed alleles but expressing GFP or YFP in cells harboring the deletion event, mice were bred with POMC-GFP or Rosa26YFP indicator mice and bred to homozygosity for the floxed allele. Mice were maintained on a 12-hr light/dark cycle with free access to water and standard mouse chow (4.25% fat, RM3; Special Diet Services) or HFD (45% fat, D12451; Research Diets) and housed in specific-pathogen free barrier facilities in individually ventilated cages of mixed genotypes. All KO and transgenic mice were studied with appropriate littermates of the three control genotypes. Mice were handled and all in vivo studies performed in accordance to the United Kingdom Animals (Scientific Procedures) Act (1986) and approved by Imperial College’s Animal Welfare and Ethical Review Body.

### Statistical Analysis

Data are expressed as mean ± SEM unless otherwise stated, and the single animal was the unit of analysis unless otherwise stated. Statistical significance was calculated at the 95% level of confidence using parametric (unpaired and paired t tests, one-way ANOVA, repeated-measures two-way ANOVA) or non-parametric (Kruskal-Wallis) tests with post hoc Bonferroni’s or Dunn’s multiple comparison analysis, where appropriate. Statistical significance was calculated from all electrophysiological recordings (responsive and non-responsive).

## Author Contributions

M.A.S., L.K., E.E.I., M.K.H., S.M.A.P., and M.W.G. generated and phenotyped the mouse lines. M.A.S. and E.E.I. performed i.c.v. feeding studies. M.A.S. performed and analyzed the electrophysiological studies. L.K., M.K.H., S.M.A.P., and A.I.C. performed and analyzed PCR analysis. L.K., A.W., and D.C. undertook and conceived western blot analysis. P.J.V. and A.V.-P. designed and conducted hyperinsulinemic-euglycemic clamp studies. M.A.S. and D.J.W. conceived and designed the study and wrote the manuscript. All authors contributed to the editing of the manuscript.

## Supplemental Information

Document S1. Supplemental Experimental Procedures, Figures S1–S4, and Tables S1 and S2

Document S2. Article plus Supplemental Information
