# Supplementary material for: Ribosomal S6K1 in POMC and AgRP Neurons Regulates Glucose Homeostasis but Not Feeding Behavior in Mice
Source: Cell Rep. 2015 Apr 9;11(3):335–43. doi: 10.1016/j.celrep.2015.03.029 (PMC4410943; doi:10.1016/j.celrep.2015.03.029)
Supplement: Document S1. Supplemental Experimental Procedures, Figures S1–S4, and Tables S1 and S2 [file mmc1.pdf]

Cell Reports

Supplemental Information

Ribosomal S6K1 in POMC and AgRP Neurons Regulates Glucose Homeostasis but Not Feeding Behavior in Mice

Mark A. Smith, Loukia Katsouri, Elaine E. Irvine, Mohammed K. Hankir, Silvia M.A. Pedroni, Peter J. Voshol, Matthew W. Gordon, Agharul I. Choudhury, Angela Woods, Antonio Vidal-Puig, David Carling, and Dominic J. Withers

Figure S1. Related to Figure 2

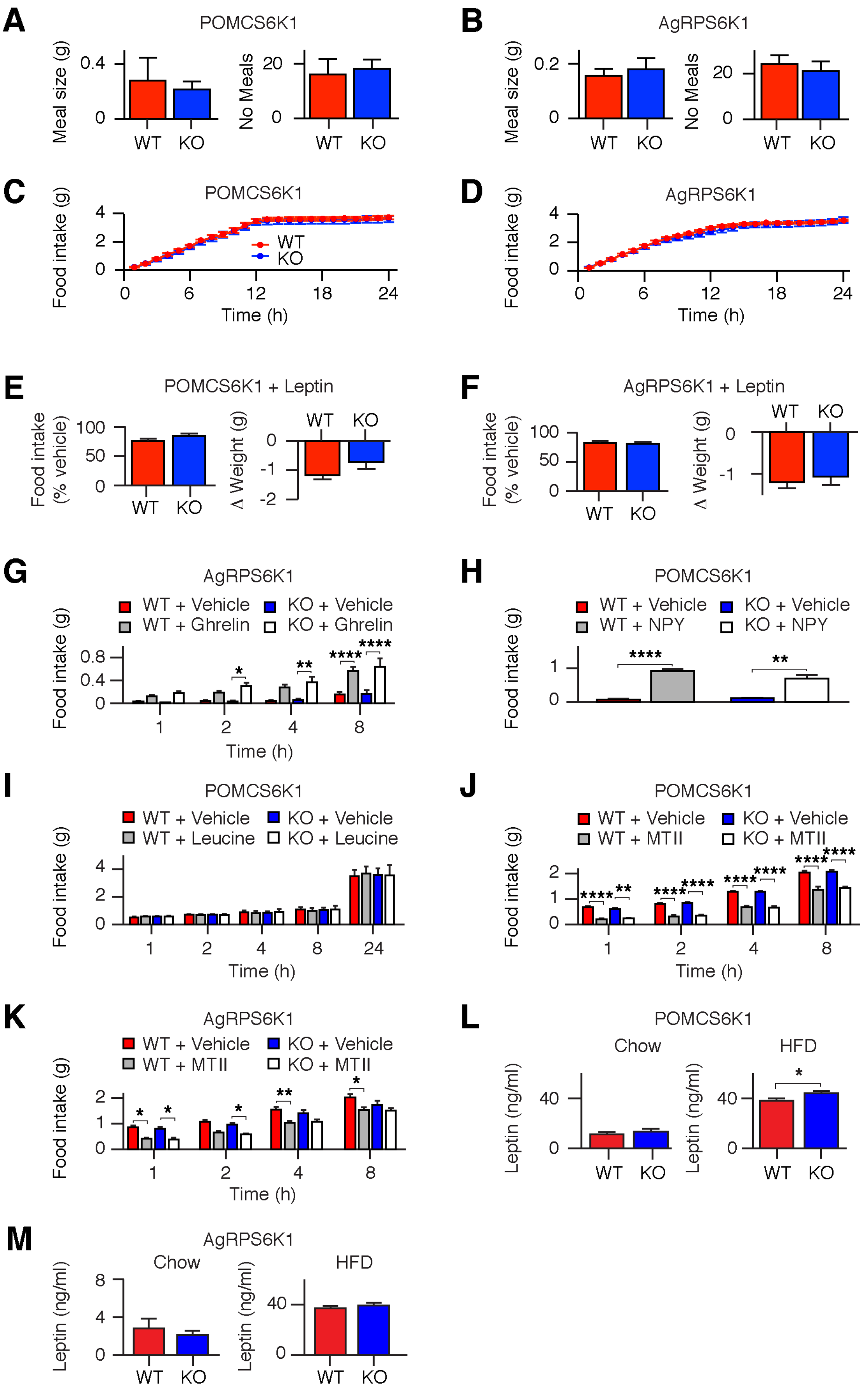

Figure S1. S6K1 is not required for the regulation of food intake

(A-D) Automated food intake monitoring of meal size (A and B, left), meal number (A and B, right) and cumulative food intake (C and D) for ad-libitum fed wild-type (WT, red bars/circles) and knockout (KO, blue bars/circles) male POMCS6K1 (A and C) and female AgRPS6K1 (B and D) mice. N=6-7 mice per genotype. Data presented is mean ± SEM.

(E and F) Leptin-induced (i.p. 0.3 mg/kg twice daily) reduction in food intake (left) and bodyweight (right) over 3 days in male POMCS6K1 (E) and AgRPS6K1 (F) WT (red bar) and KO (blue bar) mice. POMC: N=17-21; AgRP: N=17-19 mice per genotype. Data presented is mean ± SEM.

(G) Cumulative food intake for WT and KO AgRPS6K1 mice treated with vehicle (red and blue bars, respectively) or ghrelin (i.p. 5 mg/kg) (grey and white bars, respectively). N=10-12 mice per genotype. Data presented is mean ± SEM. *P<0.05, **P<0.001, ****P<0.00001.

(H) One hour food intake in WT and KO POMCS6K1 mice i.c.v. injected with vehicle (red and blue bars, respectively) or NPY (1 μg) (grey and white bars, respectively). N=3-8 mice per genotype or treatment. Data presented is mean ± SEM. **P<0.001, ****P<0.00001.

(I) Cumulative food intake in overnight fasted WT and KO POMCS6K1 mice i.c.v. injected with vehicle (red and blue bars, respectively) or leucine (2.2 μg) (grey and white bars, respectively). N=9-12 mice per genotype. Data presented is mean ± SEM.

(J and K) Cumulative food intake for WT and KO POMCS6K1 (J) and AgRPS6K1 (K) mice treated with vehicle (red and blue bars, respectively) or melanotan-II (MTII) (i.p. 2 mg/kg) (grey and white bars, respectively). N=9-12 and N=6 mice per genotype for POMCS6K1 and AgRPS6K1, respectively. Data presented is mean ± SEM. *P<0.05, **P<0.001, ****P<0.00001.

(L and M) Fasted serum leptin concentrations for WT (red bars) and KO (blue bars) mice shown in Figure 2 panels E and F. Data shown is from 34 week old POMCS6K1 (L) and AgRPS6K1 (M) mice fed on normal chow (left) or HFD (right) and shows mean ± SEM. *P<0.05.

Figure S2. Related to Figure 2

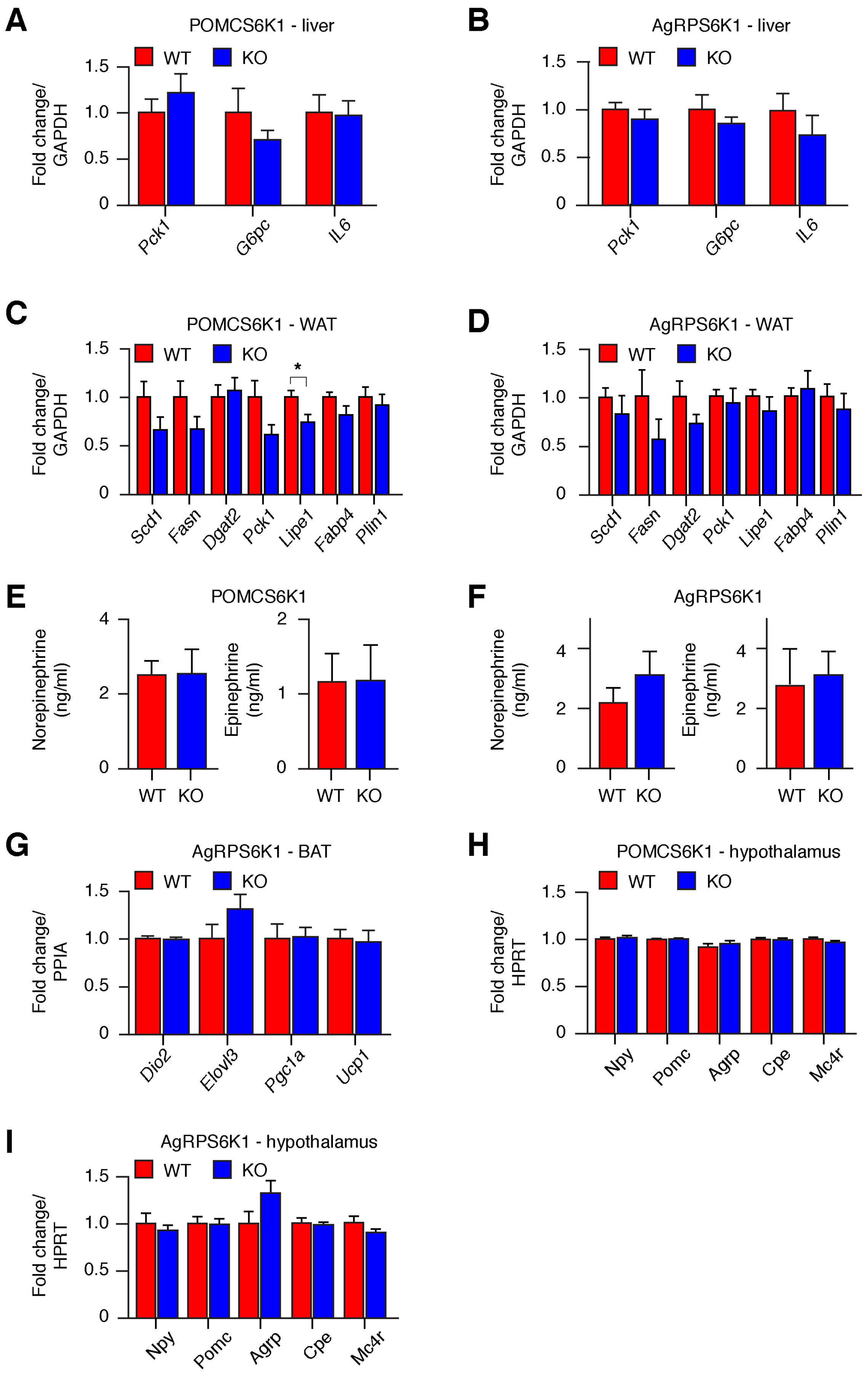

Figure S2. Gene expression in peripheral and hypothalamic tissues and urinary catecholamine concentrations

(A and B) Expression of mRNA for phosphoenolpyruvate carboxykinase-1 (*Pck1*), glucose-6-phosphatase (*G6pc*) and interleukin-6 (*IL6*) in liver of fasted wild-type (WT, red bar) and knockout (KO, blue bar) POMCS6K1 (A) or AgRPS6K1 mice (B). N=6-12 mice per genotype. Data presented is mean ± SEM.

(C and D) Expression of mRNA for stearoyl-CoA desaturase-1 (*Scd1*), fatty-acid synthase (*Fasn*), diacylglycerol o-acyltransferase 2 (*Dgat2*), *Pck1*, hormone-sensitive lipase-1 (*Lipe*), fatty-acid binding protein-4 (*Fabp4*) and perilipin-1 (*Plin1*) in white adipose tissue (WAT) of fasted WT (red bar) and KO (blue bar) POMCS6K1 (C) and AgRPS6K1 (D) mice. N=6-10 mice per genotype. Data presented is mean ± SEM. *P<0.05.

(E and F) Urinary concentrations of norepinephrine (left, N=7-13 mice per genotype) and epinephrine (right, N=4-7 mice per genotype) in fed WT (red bar) and KO (blue bar) POMCS6K1 (E) and AgRPS6K1 (F) mice. Data presented is mean ± SEM.

(G) Expression of mRNA for type-II iodothyronine deiodinase (*Dio2*), elongation of very long chain fatty acids-3 (*Elovl3*), peroxisome proliferator-activator receptor-γ coactivator-1α (*Pgc1a*) and uncoupling protein-1 (*Ucp1*) in brown adipose tissue (BAT) of fasted WT (red bar) and KO (blue bar) AgRPS6K1 mutant mice. N=9-10 mice per genotype. Data presented is mean ± SEM.

(H and I) Expression of mRNA for neuropeptide Y (*Npy*), pro-opiomelanocortin (*Pomc*), agouti-related peptide (*Agrp*), carboxypeptidase E (*Cpe*) and melanocortin-4 receptor (*Mc4r*) in the mediobasal hypothalamus of fasted WT (red bars) and KO (blue bars) POMCS6K1 (H, N=18-21 mice per genotype) or AgRPS6K1 (I, N=12 mice per genotype) mice. Data presented is mean ± SEM.

Figure S3. Related to Figure 2

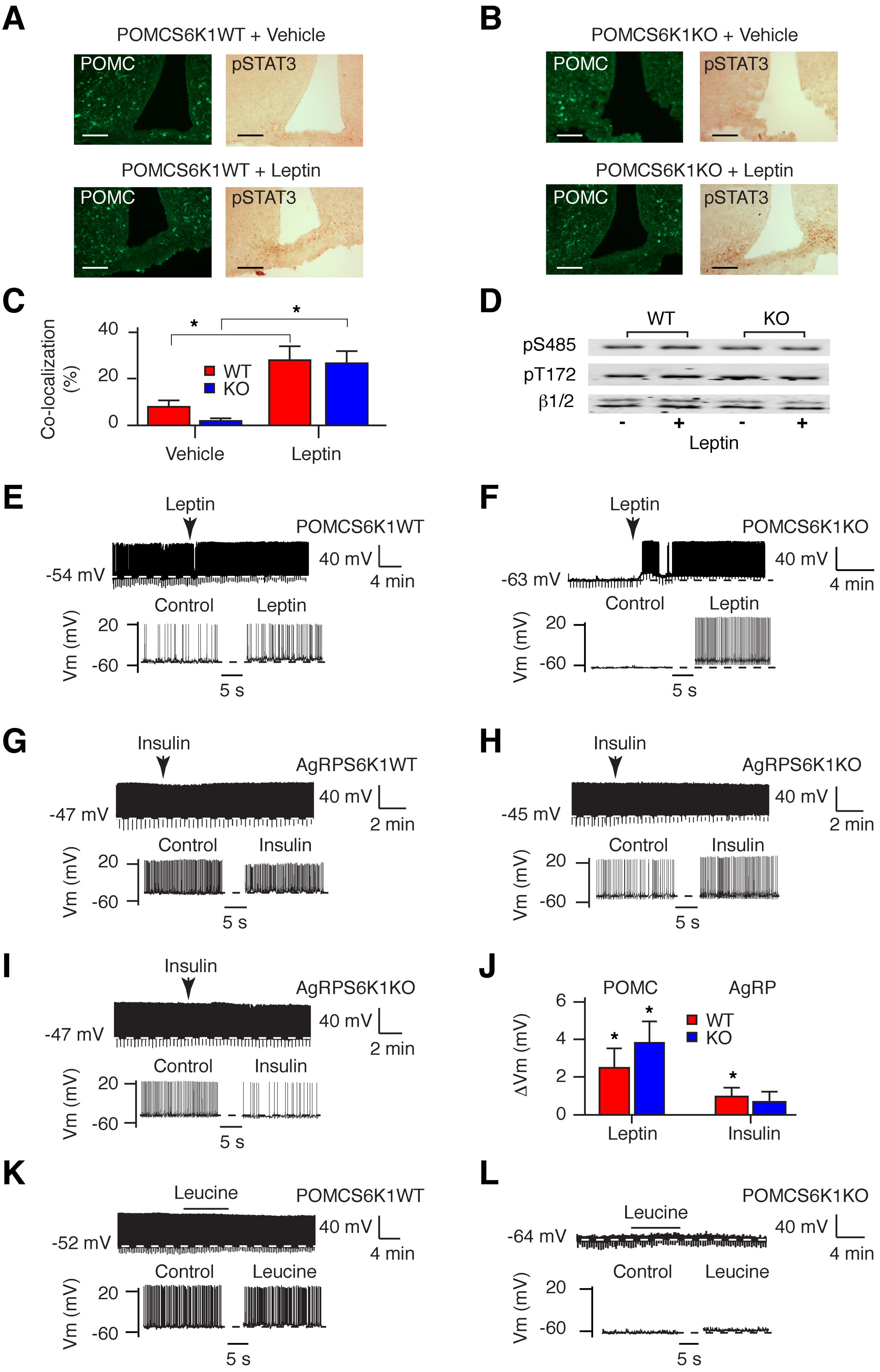

Figure S3. Leptin sensitivity is not affected by *Rps6kb1* deletion

(A and B) Representative hypothalamic sections showing immunohistochemistry for POMC (left) and phosphorylation of STAT3 (pSTAT3) (right) in POMCS6K1 wild-type (WT, A) and knockout mice (KO, B) treated with vehicle (top) or i.p. 5 mg/kg leptin (bottom). Scale bar represents 100 μm.

(C) Quantification of co-localization between POMC and pSTAT3 positive neurons. N=3-5 mice per genotype and treatment. Data presented is mean ± SEM. *P<0.05.

(D) Immunoprecipitation and western blot analysis for AMPK α1 phosphorylation at S485 and T172, and for AMPK β1/2 in hypothalamic lysates from WT and nestin-cre S6K1$^{fl/fl}$ (KO) mice treated with vehicle or i.p. 5 mg/kg leptin.

(E and F) Representative current-clamp traces from WT (E) and *Rps6kb1*-deleted (F) POMC neurons in the presence or absence of 50 nM leptin, where indicated. Expanded sections underneath are shown before and after the application of leptin.

(G-I) Representative current-clamp traces from WT (G) and *Rps6kb1*-deleted (H and I) AgRP neurons in the presence or absence of 50 nM insulin, where indicated. Expanded sections underneath are before and after the application of insulin.

(J) Bar charts showing the change in membrane potential (ΔVm) in WT (red bars) and KO (blue bars) POMC and AgRP neurons induced by leptin and insulin, respectively. Data is presented as mean ± SEM and is inclusive of non-responsive neurons. N=12-16 neurons per genotype. *P<0.05 from baseline recordings. No differences were observed between genotype.

(K and L) Representative traces from WT (K) and *Rps6kb1*-deleted (L) POMC neurons in the presence and absence of 5 mM leucine, where indicated.

Figure S4. Related to Figure 2, 3 & 4

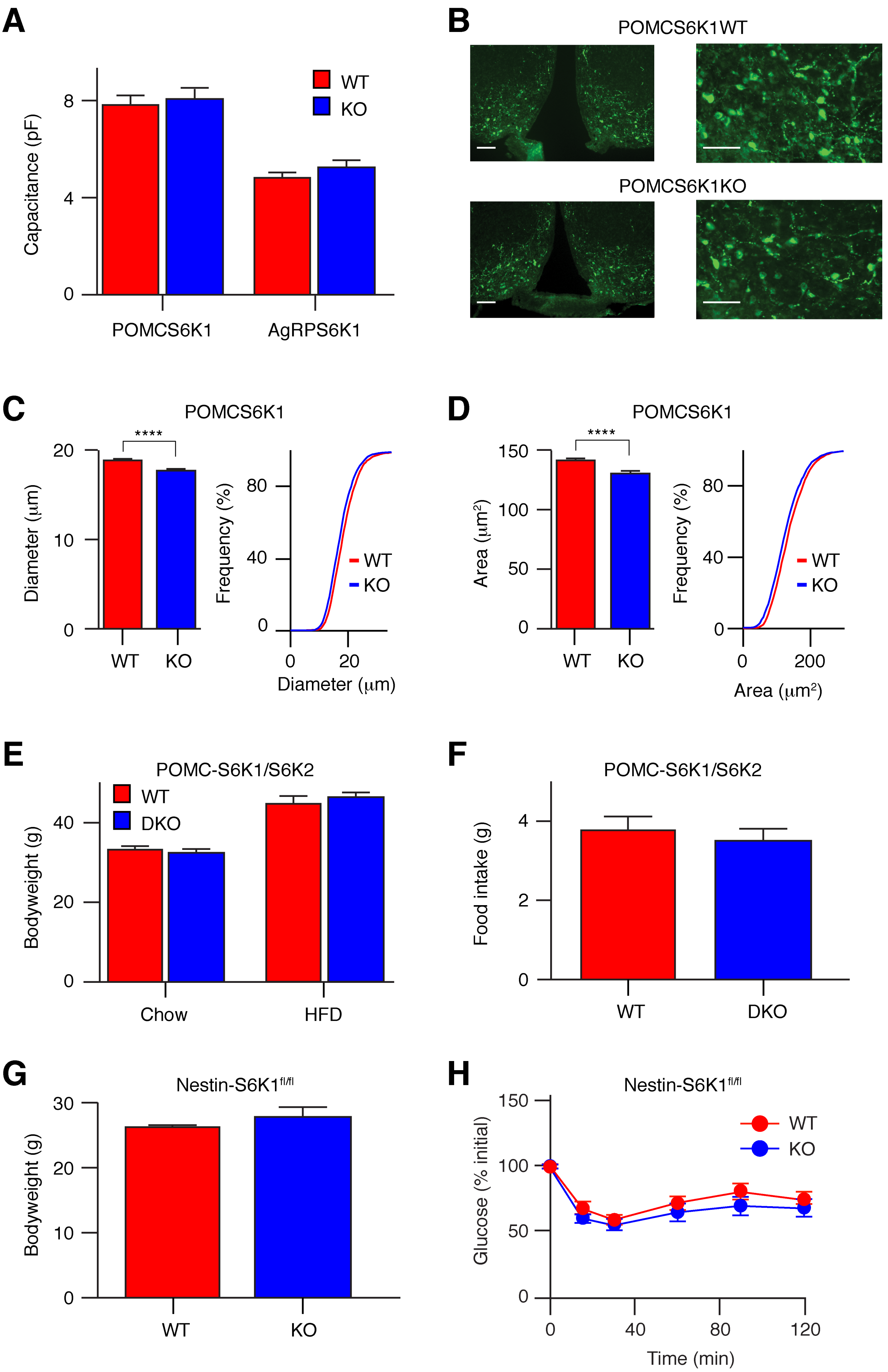

Figure S4. S6K1 in POMC neurons regulates cell body size but combined deletion of S6K1 and S6K2 in POMC neurons or deletion of S6K1 in all neurons does not alter bodyweight

(A) Whole-cell capacitance measurements from wild-type (WT, red bar) and knockout (KO, blue bar) POMCS6K1-GFP and AgRPS6K1-YFP neurons, N=22-38 neurons per genotype. Date presented is mean ± SEM.

(B) Representative low (left) and high (right) magnification images for POMC immuno-staining in WT (top) and *Rps6kb1*-deleted (bottom) POMC neurons. Scale bars represent 100 and 50 μm for low and high magnification images, respectively.

(C and D) Quantification of POMC soma diameter (C) and area (D) and calculated at equivalent coronal depths between WT and *Rps6kb1*-deleted cells. N=2471-3563 neurons from 4 mice per genotype. Data presented is mean ± SEM. ****P<0.0001.

(E) Bodyweight of male WT (red bar) and double KO (DKO, blue bar) POMC S6K1/S6K2 mice at 21 weeks on normal chow or high fat diet (HFD). N=17-24 mice per genotype. Data presented is mean ± SEM.

(F) Daily food intake in male WT (red bar) and DKO (blue bar) POMC S6K1/S6K2 mice on normal chow. N=6 mice per genotype. Data presented is mean ± SEM.

(G) Bodyweight of male WT (red bar) and KO (blue bar) nestin-cre $S6K1^{fl/fl}$ mice at 19 weeks on normal chow. N=13 mice per genotype. Data presented is mean ± SEM.

(H) Insulin-tolerance tests in WT (red circles) and KO (blue circles) male nestin $S6K1^{fl/fl}$ mice at 29 weeks of age on normal chow. N=12-13 mice per genotype. Data presented is mean ± SEM.

Table S1. CLAMS analysis of metabolism, energy expenditure and locomotion in wild-type (WT) and knockout (KO) POMCS6K1 and AgRPS6K1 mutant mice. Related to Figure 2

		POMCS6K1 Male		POMCS6K1 Female		AgRPS6K1 Male		AgRPS6K1 Female	
		WT (10)	KO (11)	WT (10)	KO (7)	WT (9)	KO (6)	WT (10)	KO (9)
VO_2 (ml/g/h)	Light	4.8±0.2	5.5±0.3	4.5±0.5	3.6±0.3	3.3±0.2	3.7±0.1	4.4±0.4	5.0±0.3
	Dark	5.6±0.3	5.6±0.3	5.2±0.6	4.0±0.4	3.9±0.3	4.4±0.2	4.7±0.4	5.2±0.3
VCO_2 (ml/g/h)	Light	4.8±0.2	5.0±0.3	4.2±0.5	3.2±0.3	3.0±0.2	3.4±0.1	4.1±0.4	4.7±0.3
	Dark	5.6±0.3	5.6±0.3	5.2±0.6	3.9±0.4	3.9±0.3	4.5±0.2	4.4±0.4	5.2±0.3
EE (Kcal/h)	Light	0.71±0.03	0.74±0.04	0.51±0.06	0.40±0.03	0.49±0.04	0.56±0.01	0.54±0.04	0.51±0.02
	Dark	0.85±0.05	0.82±0.05	0.60±0.08	0.46±0.04	0.59±0.05	0.68±0.02	0.57±0.05	0.54±0.03
RER (VCO_2/VO_2)	Light	0.87±0.01	0.91±0.02	0.91±0.02	0.92±0.01	0.90±0.01	0.92±0.01	0.91±0.01	0.92±0.01
	Dark	0.95±0.02	0.94±0.03	0.99±0.01	0.97±0.02	1.01±0.01	1.02±0.01	0.93±0.01	0.95±0.01
x-axis (count/min)	Light	16.2±2.4	22.6±3.5	21.3±2.8	19.1±2.4	17.6±2.8	12.8±2.6	28.7±3.2	29.8±3.1
	Dark	55.9±9.6	53.5±11.6	64.1±8.2	56.5±13.6	39.9±5.3	35.3±5.9	44.7±5.0	46.4±5.2
z-axis (count/min)	Light	1.1±0.3	1.2±0.2	2.3±0.7	2.4±0.4	1.2±0.2	1.0±0.2	9.7±3.9	11.4±2.9
	Dark	6.0±1.2	4.3±1.2	10.3±2.2	10.1±1.4	3.8±0.7	4.2±0.5	13.4±4.2	17.3±3.9

Data expressed as mean ± SEM during the light and dark phases. Number of mice per genotype shown in parenthesis. EE, energy expenditure; RER, respiratory exchange ratio.

Table S2. Blood glucose and serum insulin concentrations in wild-type (WT) and knockout (KO) POMCS6K1 and AgRPS6K1 mutant mice. Related to Figure 2

		POMCS6K1 Male		POMCS6K1 Female		AgRPS6K1 Male		AgRPS6K1 Female	
		WT	KO	WT	KO	WT	KO	WT	KO
Fed glucose (mM)	8 week old, Chow	8.8±0.3 (24)	9.0±0.3 (21)	8.7±0.3 (24)	8.7±0.2 (24)	10.5±0.3 (28)	10.2±0.3 (20)	9.3±0.2 (29)	9.6±0.3 (22)
	26 week old, Chow	9.2±0.3 (18)	9.6±0.2 (22)	7.9±0.3 (19)	8.2±0.3 (22)	11.0±0.3 (33)	10.3±0.3 (23)	10.4±0.4 (29)	9.5±0.3 (25)
	26 week old, HFD	11.4±0.5 (20)	10.7±0.3 (21)	8.5±0.2 (22)	8.6±0.2 (21)	12.9±0.5 (26)	12.7±0.5 (19)	9.9±0.3 (25)	9.7±0.3 (15)
Fasted glucose (mM)	8 week old, Chow	5.3±0.4 (15)	4.8±0.2 (22)	5.2±0.4 (17)	5.8±0.4 (19)	6.1±0.1 (30)	6.1±0.2 (23)	6.0±0.2 (27)	6.2±0.2 (18)
	26 week old, Chow	5.7±0.4 (18)	5.6±0.3 (22)	6.4±0.3 (19)	6.5±0.4 (22)	6.4±0.1 (31)	6.2±0.2 (23)	4.9±0.1 (31)	4.9±0.1 (25)
	26 week old, HFD	7.1±0.4 (20)	7.4±0.3 (21)	5.6±0.3 (22)	5.6±0.3 (21)	7.4±0.3 (25)	7.6±0.3 (19)	7.2 ±0.4 (24)	7.3±0.4 (15)
Fasted insulin (ng/ml)	Chow	0.53±0.11 (16)	0.79±0.17 (22)	0.31±0.05 (16)	0.38±0.05 (15)	0.38±0.03 (32)	0.49±0.11 (21)	0.58±0.08 (29)	0.70±0.23 (24)
	HFD	0.81±0.15 (19)	0.93±0.15 (21)	0.50±0.08 (15)	0.52±0.14 (17)	2.0±0.2 (26)	1.7±0.14 (19)	0.66±0.11 (24)	0.75±0.09 (13)

Data expressed as mean ± SEM. Number of mice per genotype shown in parenthesis. HFD, high fat diet

SUPPLEMENTAL EXPERIMENTAL PROCEDURES

Genotyping

For detection of Cre-mediated excision of exons 3 and 4 of *Rps6kb1* in the hypothalami of AgRPS6K1KO and POMCS6K1KO mice, genomic DNA was isolated from the hypothalamus, cortex, liver and skeletal muscle as previously described (Choudhury et al., 2005). The generation of a ~500 bp DNA product following PCR with primers TCCACCCACCAGTAAAGAGC and CCTCAGTCTCCTGAGTGTTAAGG is indicative of the floxed allele, whereas deletion is denoted by a ~450 bp band generated by primers TCCACCCACCAGTAAAGAGC and ACAAGAGGGCCAGTTGATGG.

Metabolic and food intake studies

Studies were performed in the animal's home cage unless indicated. Bodyweights from group-housed mice were measured weekly at 9-10am until 26 weeks of age. Glucose (i.p. 2 g/kg) and insulin (i.p. 0.75 U/kg) -tolerance tests were performed at 8 and 26 weeks of age, as previously described (Al-Qassab et al., 2009; Choudhury et al., 2005). At 34 weeks of age, fed mice were weighed prior to EchoMRI analysis of body composition. Fasted tail blood was analyzed for serum leptin (Millipore) and insulin (Crystal Chem.) by ELISA and for FFA (WAKO Chem.) and triglycerides (Sigma Aldrich). Serum corticosterone levels were determined by ELISA (Immunodiagnostics Systems) from fed mice. For food intake studies, mice were group housed until 10 weeks of age and then singly housed. Mice were allowed to acclimatize for 2 weeks and periodically fasted overnight to acclimatize them. Ad-libitum food intake was measured over 3 consecutive days and for 24 h following an overnight fast. Food intake was measured from singly housed mice injected with either vehicle or leptin (i.p. 0.3 mg/kg) at 9am and again at 6pm for 3 consecutive days. Food intake was also measured in fed mice for 8-24 hours following the injection of ghrelin (i.p. 5 mg/kg) or melanotan-II (MTII, i.p. 2 mg/kg) at 9am. Stainless steel cannulae (Plastics One Inc.) were stereotaxically implanted into the lateral cerebral ventricle so that the cannula tip was 0.4 mm posterior to bregma, 1.0 mm lateral to the midline and -2.1 mm ventral to the surface of the skull. Post-

surgery, the mice were singly housed, left for one week to recover and then habituated to overnight fasts for 2 weeks. Following an overnight fast, mice were injected with 0.5 μl of artificial cerebrospinal fluid (aCSF) or leucine (i.c.v. 2.2 μg) and food intake monitored over a 24 h period. All injections were performed with a 31-gauge stainless steel injector which projected 0.5 mm below the tip of the cannula. Following the infusion, the injector was left in place for an additional 30 s to allow the drugs to diffuse away from the cannula tip. Treatments, with either vehicle or drug, were crossed-over following a 1 week wash-out period. Correct cannula placement was confirmed at the end of the study by an increase in food intake after i.c.v. administration of NPY (1 μg). Mice were singly housed and habituated in CLAMS cages (Columbus Instruments) for 1-2 days prior to assessment of locomotion and energy expenditure over the subsequent 2 days.

Automated food intake monitoring

An episodic food intake monitoring apparatus (BioDAQ, Research Diets, Inc.) was used to assess feeding patterns in singly housed mice on normal chow. Food intake was measured and averaged from 5 consecutive days to obtain 24 h food intake kinetics. The BioDAQ system weighs the hopper with food (± 10 mg) every second and uses an algorithm to determine feeding bouts (changes in stable weight before and after a bout). Meals consist of one or more bouts separated by an inter-meal interval of 300 s with a minimum meal size of 20 mg.

Quantitative RT-PCR analysis

Tissues were lysed and homogenized in TRIzol reagent (Ambion) and total RNA was isolated using the RNeasy mini kit (Qiagen). First-strand cDNA was generated using Taqman reverse transcription reagents (Applied Biosystems) and qPCR was performed using Taqman universal PCR mastermix in a 7900HT real-time PCR system (Applied Biosystems). mRNA quantities were normalized to *Hprt*, *Ppia* or *Gapdh* after determination by the comparative Ct method. Primers used were: *Abcc8* (Mm00803450), *Agrp*

(Mm00475829_g1), *Cpe* (Mm00516341_m1) *Dgat2* (Mm00499536_m1), *Dio2* (Mm00515664_m1), Elov3 (Mm00468164_m1) *Fabp4* (Mm00445878_m1), *Fasn* (Mm00662319_m1), *Gabra1* (Mm00439046_m1), *Gabra2* (Mm00433435_m1), *Gabra3* (Mm01294271_m1), *Gabra5* (Mm00621092_m1), *Gapdh* (Mm99999915_g1), *Gck* (Mm00439129_m1), *Hprt* (Mm00446968_m1), *Lipe* (Mm00495359_m1), *IL6* (Mm00446190-m1), *Kcnj11* (Mm00440050_m1), *Mc4r* (Mm00457483_s1), *Npy* (Mm00445771_m1), *Pck1* (Mm01247058_m1), *Pgc1a* (Mm00447183_m1), *Plin1* (Mm00558672_m1), *Pomc* (Mm00435874_m1), *Ppia* (Mm03302254_g1), *Scd1* (Mm00772290_m1), *Ucp1* (Mm01244861_m1).

Hypothalamic immunohistochemistry

Immunohistochemistry was performed as previously described (Al-Qassab et al., 2009; Choudhury et al., 2005). Fasted mice were injected with leptin (i.p. 5 mg/kg) and perfused with paraformaldehyde (4% w/v). Arcuate sections were incubated with rabbit anti-POMC precursor (1:1000; Phoenix Pharmaceuticals Inc.) and detection performed using a secondary antibody coupled to Alexa-Fluor-488. After extensive washing, slices were incubated with a rabbit anti-pSTAT3 (Tyr705) antibody (1.1000, Cell Signaling) which was detected using an ABC detection kit (Vector labs). Fluorescent images were taken with an epifluorescence microscope fitted with a color digital camera. Mice expressing GFP (POMC-GFP mouse) or YFP (AgRPCre-YFP) were fixed and arcuate sections incubated with a rabbit p70 S6 kinase (49D7, Cell Signaling) primary antibody (1:200) followed by a secondary antibody coupled to Alexa-Fluor-594. POMC neuronal measurements were counted from labeled cells using ImageJ software.

Electrophysiology

Hypothalamic coronal slices (350 μm) were cut from aged (3 or 7 month old) matched transgenic mice expressing POMCCre/POMC-GFP or AgRPCre/Rosa26YFP with or without floxed *Rps6bk1*. Slices were maintained at room temperature (22-25ºC) in an external

solution containing (in mM) NaCl 125, KCl 2.5, NaH_2PO_4 1.25, $NaHCO_3$ 25, $CaCl_2$ 2, $MgCl_2$ 1, D-glucose 10, D-mannitol 15, equilibrated with 95% O_2, 5% CO_2, pH 7.4. POMC and AgRP neurons were visualized in the arcuate nucleus by the expression and excitation of GFP and YFP, respectively. Whole-cell current-clamp (I_{fast}) recordings were made at ~35°C using borosilicate glass pipettes (4-8 MΩ) containing (in mM) Kgluconate 130, KCl 10, EGTA 0.5, NaCl 1, $CaCl_2$ 0.28, $MgCl_2$ 3, Na_2ATP 3, GTP 0.3, phosphocreatine 14 and HEPES 10 (pH 7.2), as previously described (Al-Qassab et al., 2009; Choudhury et al., 2005). Following a minimum of 10 min of stable recording, hormones were applied for 2-3 min using a broken tipped pipette (~3 μm) positioned above the recording neuron. Stock reagents were diluted (≥1000 fold) in a modified external solution with $NaHCO_3$ replaced with HEPES (10 mM, pH 7.4). Stocks of recombinant leptin (R&D Systems) and insulin (Novo-Nordisk Inc.) were diluted in HEPES-buffered external solution. All other reagents were purchased from Sigma-Aldrich. External glucose was replaced with mannitol to maintain osmolarity and biophysical properties were obtained from separate recordings in different glucose concentrations. Neurons were voltage-clamped at -70 mV using a modified internal solution in which Kgluconate was replaced with CsCl (130 mM). Slices were bathed in tetrodotoxin (1 μM) and (+)-bicuculline (20 μM) for miniature excitatory currents (mEPSC) or NBQX (5 μM, 2,3-dihydroxy-6-nitro-7-sulfamoyl-benzo[f]quinoxaline-2,3-dione) and AP5 (50 μM (2*R*)-amino-5-phosphonovaleric acid; (2*R*)-amino-5-phosphonopentanoate) for miniature inhibitory currents (mIPSC). Note that that there were no statistical differences in the excitable properties of POMC neurons in young and old mice (Vm; 3 month, WT: -48.6±1.3 vs KO: -53.3±1.6 mV, n=24-29; 7 month, WT: -49.6±2.0 vs KO: -54.1±1.3 mV, n=9-16).

Hyperinsulinemic-euglycemic clamps studies

Clamps were conducted as previously described (Voshol et al., 2001). Animals were anesthetized by intraperitoneal injection of a combination of 6.25 mg/kg acetylpromazine, 6.25 mg/kg midazolam and 0.31 mg/kg fentanyl. An infusion needle was placed into the tail

vein and D-[^{3}H] glucose (specific activity: 10-20Ci (370-740GBq)/mmol) was infused at a rate of 0.006 MBq/min for 60 min to achieve steady-state levels. Thereafter, insulin (Actrapid; Novo Nordisk) was infused at a constant rate of 0.09 mU/min after a bolus dose of 3.3 mU and D-[^{3}H]-glucose was continued at a rate of 0.006 MBq/min. A variable infusion of 12.5% D-glucose was used to maintain blood glucose at euglycemic (basal) levels. Blood glucose was measured with an AlphaTRAK glucometer (Abbott Animal Health) every 5-10 minutes and glucose infusion adjusted accordingly. After 50 minutes from the start of the insulin infusion, ^{14}C-2-Deoxy-glucose-phosphate (Specific Activity: 250-350mCi (9.25-13.0GBq)/mmol) was administered i.v. to assess tissue-specific glucose uptake. Steady-state was reached after 90 minutes and blood samples were taken at 10 minutes intervals over 30 minutes to determine steady-state levels of [^{3}H]-glucose. Mice were then killed by cervical dislocation and the organs removed and frozen. Haematocrit was measured at baseline and after the clamp (no significant changes were observed between each genotype and their littermate controls, data not shown). To measure plasma [^{3}H]-glucose, proteins were precipitated with trichloroacetic acid (final concentration 10%), centrifuged, and supernatant dried and re-suspended in water. The samples were counted using scintillation counting (Hidex Scintilation counter, LabLogic). Tissue samples were homogenized (~5-10% wet wt/vol, depending on tissue) in 0.5% percholic acid, centrifuged, supernatants neutralized, and ^{14}C-2-Deoxy-glucose-phosphate precipitated using BaOH/$ZnSO_4$. Total and precipitated counts of supernatants were subtracted and plasma ^{14}C-2-Deoxy-glucose-phosphate counts were used to calculate tissue specific uptake. Protein content in homogenates was performed using DC protein assay (BioRad). The glucose turnover rate (µmol/min) was calculated during the basal period and under steady-state clamp conditions as the rate of tracer infusion (dpm/min) divided by the plasma specific activity of [^{3}H] glucose (dpm/µmol). The hyperinsulinemic hepatic glucose production was calculated as the difference between the tracer-derived rate of glucose appearance and the glucose infusion rate.

Western blot analysis

Tissues were removed and homogenized in lysis buffer (50 mM Tris pH 7.4, 150 mM NaCl, 1 mM EDTA, 1% w/v Triton X-100) supplemented with Roche complete protease inhibitor cocktail and phosphatase inhibitors (1 mM sodium orthovadanate, 5 mM sodium fluoride and 2 mM β-glycerophosphate). 20-100 μg of total protein homogenates were run on 15% SDS-PAGE gels, transferred to PVDF membranes and blotted with antibodies against p70 S6 Kinase (1:3000, #2708 Cell Signaling), total S6 Ribosomal Protein (1:1000, #2217 Cell Signaling), phospo-S6 Ribosomal Protein Ser240/244 (1:1000, #2708 Cell Signaling), and tubulin as a loading control (1:10,000, #T5293 Sigma). Detection was performed using enhanced chemiluminescence (Luminata Crescento, Millipore) and exposed on films. The intensity of the bands was quantified by densitometry using ImageJ software and normalized to either β-tubulin or total S6 ribosomal protein. For AMPK studies, hypothalamic tissue was homogenized in buffer (50 mM Tris-HCl pH 8.4 (at 4°C), 50 mM NaF, 5 mM NaPP, 0.25 M sucrose (or mannitol), 1 mM EDTA, 1 mM benzamidine, 0.1 mM PMSF, 1 mM DTT) and supernatants were either western blotted or used for immunoprecipitation (500 μg). Lysates were pre-cleared using protein G sepharose and then immunoprecipitated using a sheep antibody specific for AMPK α1. The immune-complexes were washed and run on SDS PAGE gels (Novex precast Tris/Bis gels). Antibodies used for blotting AMPKβ1/2, AMPKp-T172 and AMPKpS485/491 were all from Cell Signaling.

General experimental approaches

Where possible, investigators were blinded to the genotype of both study animals and that of tissue and blood samples. For experiments involving treatments, mice were randomized by genotype to study groups or a cross-over design was used where indicated and study cohorts were matched for initial bodyweight where appropriate. Treatments were administered in random order. All metabolic studies were replicated in at least 2 independent cohorts. Study cohort sizes were determined by power calculations based on our previous data in mice with targeted hypothalamic mutations. In electrophysiological experiments, a

typical mouse allowed 1-5 recordings but observations were repeated on at least 4 different mice.

SUPPLEMENTAL REFERENCES

Al-Qassab, H., Smith, M.A., Irvine, E.E., Guillermet-Guibert, J., Claret, M., Choudhury, A.I., Selman, C., Piipari, K., Clements, M., Lingard, S., *et al.* (2009). Dominant role of the p110beta isoform of PI3K over p110alpha in energy homeostasis regulation by POMC and AgRP neurons. Cell Metab *10*, 343-354.

Choudhury, A.I., Heffron, H., Smith, M.A., Al-Qassab, H., Xu, A.W., Selman, C., Simmgen, M., Clements, M., Claret, M., Maccoll, G., *et al.* (2005). The role of insulin receptor substrate 2 in hypothalamic and beta cell function. J Clin Invest *115*, 940-950.

Claret, M., Smith, M.A., Knauf, C., Al-Qassab, H., Woods, A., Heslegrave, A., Piipari, K., Emmanuel, J.J., Colom, A., Valet, P., *et al.* (2011). Deletion of Lkb1 in pro-opiomelanocortin neurons impairs peripheral glucose homeostasis in mice. Diabetes *60*, 735-745.

Voshol, P.J., Jong, M.C., Dahlmans, V.E., Kratky, D., Levak-Frank, S., Zechner, R., Romijn, J.A., and Havekes, L.M. (2001). In muscle-specific lipoprotein lipase-overexpressing mice, muscle triglyceride content is increased without inhibition of insulin-stimulated whole-body and muscle-specific glucose uptake. Diabetes *50*, 2585-2590.
