# Supplementary material for: Ribosomal S6K1 in POMC and AgRP Neurons Regulates Glucose Homeostasis but Not Feeding Behavior in Mice
Source: Cell Rep. 2015 Apr 9;11(3):335–43. doi: 10.1016/j.celrep.2015.03.029 (PMC4410943; doi:10.1016/j.celrep.2015.03.029)
Supplement: Document S2. Article plus Supplemental Information [file mmc2.pdf]

Cell Reports

Report

Ribosomal S6K1 in POMC and AgRP Neurons Regulates Glucose Homeostasis but Not Feeding Behavior in Mice

Graphical Abstract

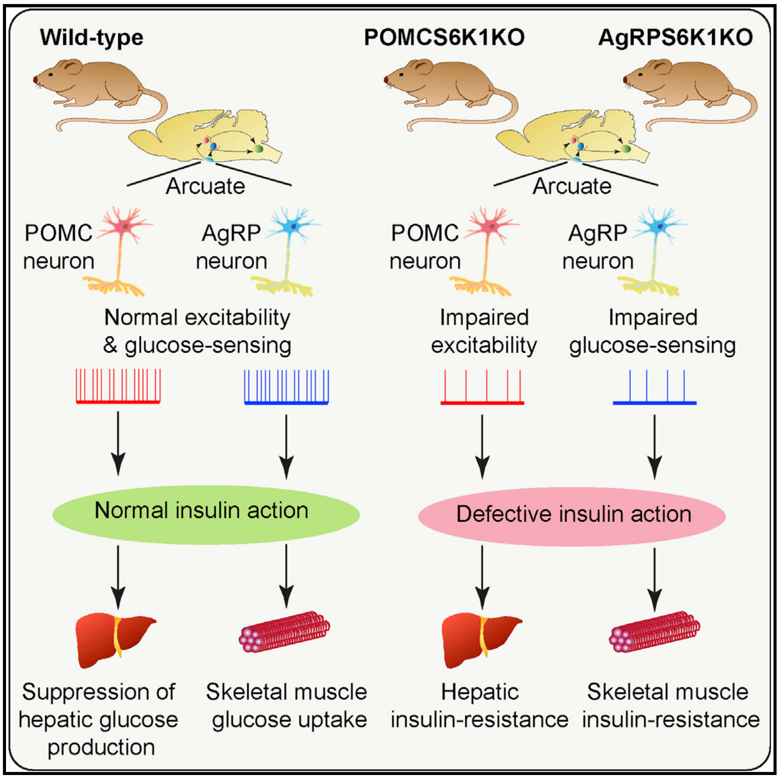

Highlights

- Mice lacking S6K1 in POMC and AgRP neurons have impaired glucose homeostasis
- S6K1-deficient POMC and AgRP neurons have altered excitability
- S6K1 in POMC and AgRP neurons does not regulate food intake and bodyweight

Authors

Mark A. Smith, Loukia Katsouri, ..., David Carling, Dominic J. Withers

Correspondence

mark.smith@imperial.ac.uk (M.A.S.), d.withers@imperial.ac.uk (D.J.W.)

In Brief

POMC and AgRP neurons regulate systemic energy homeostasis. Smith et al. demonstrate that S6K1 signaling in these cells regulates neuronal excitability and peripheral glucose homeostasis in mice. However, POMC and AgRP S6K1 signaling does not play a major role in feeding behavior and the control of bodyweight.

Smith et al., 2015, Cell Reports *11*, 335–343
April 21, 2015 ©2015 The Authors
http://dx.doi.org/10.1016/j.celrep.2015.03.029

Cell Reports
Report

OPEN ACCESS
CellPress

Ribosomal S6K1 in POMC and AgRP Neurons Regulates Glucose Homeostasis but Not Feeding Behavior in Mice

Mark A. Smith,[1,*] Loukia Katsouri,[1] Elaine E. Irvine,[1] Mohammed K. Hankir,[1,4] Silvia M.A. Pedroni,[1] Peter J. Voshol,[2,5] Matthew W. Gordon,[1] Agharul I. Choudhury,[1] Angela Woods,[3] Antonio Vidal-Puig,[2] David Carling,[3] and Dominic J. Withers[1,*]

[1]Metabolic Signalling Group, Medical Research Council Clinical Sciences Centre, Imperial College London, Hammersmith Campus, London W12 0NN, UK

[2]University of Cambridge, Metabolic Research Laboratories, MRC Metabolic Diseases Unit, Wellcome Trust-MRC Institute of Metabolic Science, Level 4, Box 289, Addenbrooke's Hospital, Cambridge CB2 0QQ, UK

[3]Cellular Stress Group, Medical Research Council Clinical Sciences Centre, Imperial College London, Hammersmith Campus, London W12 0NN, UK

[4]Present address: Integrated Research and Treatment Centre for Adiposity Diseases, Universität(s) Medizin Leipzig, Liebigstraße 21, 04103 Leipzig, Germany

[5]Present address: Louis Bolk Institute, Hoofdstraat 24, 3972 LA Driebergen, the Netherlands

*Correspondence: mark.smith@imperial.ac.uk (M.A.S.), d.withers@imperial.ac.uk (D.J.W.)

http://dx.doi.org/10.1016/j.celrep.2015.03.029

This is an open access article under the CC BY license (http://creativecommons.org/licenses/by/4.0/).

SUMMARY

Hypothalamic ribosomal S6K1 has been suggested as a point of convergence for hormonal and nutrient signals in the regulation of feeding behavior, bodyweight, and glucose metabolism. However, the long-term effects of manipulating hypothalamic S6K1 signaling on energy homeostasis and the cellular mechanisms underlying these roles are unclear. We therefore inactivated S6K1 in pro-opiomelanocortin (POMC) and agouti-related protein (AgRP) neurons, key regulators of energy homeostasis, but in contrast to the current view, we found no evidence that S6K1 regulates food intake and bodyweight. In contrast, S6K1 signaling in POMC neurons regulated hepatic glucose production and peripheral lipid metabolism and modulated neuronal excitability. S6K1 signaling in AgRP neurons regulated skeletal muscle insulin sensitivity and was required for glucose sensing by these neurons. Our findings suggest that S6K1 signaling is not a general integrator of energy homeostasis in the mediobasal hypothalamus but has distinct roles in the regulation of glucose homeostasis by POMC and AgRP neurons.

INTRODUCTION

Obesity and its associated diseases such as type 2 diabetes and cancer are major causes of morbidity and premature mortality (Kopelman, 2000). The CNS orchestrates food intake, nutrient storage, energy expenditure, and related behaviors (Morton et al., 2014; Williams and Elmquist, 2012). Key neuronal populations have been identified that respond to both hormonal and nutrient signals that encode information about the internal metabolic milieu and environmental factors such as diet and stress (Williams and Elmquist, 2012). For example, anorexigenic pro-opiomelanocortin (POMC) and orexigenic agouti-related peptide (AgRP)-expressing neurons in the hypothalamic arcuate nucleus coordinately regulate food intake and energy expenditure as well as peripheral tissue glucose homeostasis and energy partitioning (Varela and Horvath, 2012).

A significant body of work has focused on identification of common intracellular signaling pathways that respond to a range of metabolic signals and may couple hormone and nutrient sensing by neurons to the regulation of organismal metabolism. One such pathway is the mechanistic target of rapamycin (mTOR) signaling network (André and Cota, 2012; Blouet and Schwartz, 2010; Haissaguerre et al., 2014). mTOR exists in two distinct multimolecular complexes, mTORC1 and mTORC2, which are thought to subserve distinct cellular roles (Laplante and Sabatini, 2012). mTORC1 is responsive to a range of factors, including growth factors, amino acids, energy status, and oxygen and has been implicated in the regulation of protein synthesis, lipogenesis, nucleic acid synthesis, and other processes (Laplante and Sabatini, 2012). mTORC1 regulates, through phosphorylation, a number of downstream effector proteins, the best characterized of which are p70 ribosomal S6 protein kinase-1 (S6K1) and eukaryotic translation initiation factor-4E (eIF4E)-binding protein-1 (4E-BP1) (Laplante and Sabatini, 2012). S6K1 has been implicated in ribosomal biogenesis and translational regulation as well as the control of cell size, gene transcription, and the feedback regulation of insulin signaling (Magnuson et al., 2012).

Hypothalamic mTORC1 signaling and S6K1 in particular have been linked to the regulation of energy homeostasis (Blouet and Schwartz, 2010; Haissaguerre et al., 2014). S6K1 is expressed in the hypothalamus, including in POMC and AgRP neurons where it is sensitive to nutrient status (Cota et al., 2006). Hormonal

regulators of feeding behavior and bodyweight such as leptin, insulin, and ghrelin modulate hypothalamic S6K1 signaling (Zhang et al., 2011). Pharmacological inhibition of mTORC1 in the hypothalamus using rapamycin blocks the anorectic effect of leptin, leucine, and ghrelin (Cota et al., 2006; Martins et al., 2012), and mice globally lacking S6K1 are resistant to the anorectic effects of leptin (Cota et al., 2008). Adenovirally mediated acute overexpression of a constitutively active S6K1 in the mediobasal hypothalamus (MBH) of rats suppresses food intake, lowers weight gain, and improves insulin sensitivity (Blouet et al., 2008), while dominant-negative S6K1 increases food intake and weight gain, suggesting bidirectional regulation of energy homeostasis by S6K1 (Blouet et al., 2008). Mediobasal hypothalamic S6K1 may regulate peripheral glucose metabolism although, in contrast to the findings described above, constitutive activation of S6K1 results in hepatic insulin resistance (Ono et al., 2008). These observations suggest that mTORC1 and S6K1 in particular may play a key integrative role in hypothalamic nutrient and hormonal sensing and the regulation of energy homeostasis.

Despite these findings, a number of issues remain unclear with respect to the role of S6K1 in the hypothalamus. For example, the use of acute non-targeted viral systems to manipulate S6K1 signaling has not permitted long-term studies on energy balance and glucose homeostasis or revealed any specific contribution of POMC and AgRP neurons. The effects of hypothalamic S6K1 on peripheral glucose metabolism also require clarification (Blouet et al., 2008; Ono et al., 2008). Finally, there is little information on how altered hypothalamic S6K1 signaling might mechanistically regulate neuronal function and energy homeostasis. We therefore generated mice lacking S6K1 (*Rps6kb1*) specifically in either POMC or AgRP neurons. In contrast to published studies, we found a minimal role for S6K1 in the long-term regulation of food intake and bodyweight. S6K1 in POMC and AgRP neurons, however, played a key role in neuronal excitability and the regulation of peripheral glucose homeostasis.

RESULTS

Mice with Deletion of S6K1 in POMC and AgRP Neurons

Mice were generated on a C57Bl/6 background with exons 3 and 4 of *Rps6kb1* flanked by loxP sites (Figure 1A). When bred to homozygosity, *Rps6kb1* floxed (S6K1$^{fl/fl}$) mice were phenotypically indistinguishable from WT animals and displayed normal *Rps6kb1* expression in the absence of cre-recombinase (data not shown). In mice with either germ-line deletion or with global neuronal deletion (using either nestin-cre or synapsin-cre transgenic mice), western blotting confirmed complete loss of S6K1 in all tissues or specifically in the CNS, respectively (Figure 1B; data not shown). Phosphorylated S6 was significantly reduced in whole-brain lysates from nestin-cre S6K1$^{fl/fl}$ mice (Figure 1C). S6K1$^{fl/fl}$ mice were subsequently bred with mice expressing cre-recombinase driven by *Pomc* or *Agrp* promoters (POMCS6K1 or AgRPS6K1, respectively), and the recombination event was restricted to the hypothalamus in both knockout (KO) lines (Figures 1D and 1E). S6K1 immunoreactivity was co-localized with WT fluorescently labeled POMC and AgRP arcuate neurons, but expression was absent in *Rps6kb1*-deleted neurons (Figures 1F and 1G). POMC cre-recombinase mice also drive deletion in pituitary corticotrophs, but corticosterone levels were equivalent between WT and POMCS6K1-KO mice (WT, 41.3 ± 7.7 versus KO, 45.3 ± 7.7 ng/ml, n = 4–6).

Assessment of Feeding and Bodyweight Phenotypes in POMCS6K1KO and AgRPS6K1KO Mice

Food intake, ad libitum and in response to a fast, was unaltered in POMCS6K1KO and AgRPS6K1KO mice on chow (Figures 2A–2D), and average meal size, meal number, or feeding pattern over a 24-hr period were not different (Figures S1A–S1D). Both mutant lines responded normally to leptin and AgRPS6K1KO mice displayed an unaltered response to ghrelin (Figures S1E–S1G). Central neuropeptide Y (NPY, 1 μg) evoked rapid feeding, but administration of leucine (2.2 μg) to the same animals did not suppress feeding in both WT and POMCS6K1KO mice (Figures S1H and S1I). POMCS6K1KO and AgRPS6K1KO mice responded normally to the effects of the melanocortin agonist MTII (Figures S1J and S1K). As decreased melanocortin receptor signaling can enhance adiposity in the absence of hyperphagia (Nogueiras et al., 2007), we examined long-term bodyweight regulation on chow and high-fat diet (HFD). Up to 34 weeks of age, there were no differences in bodyweight, fat mass, and serum leptin concentrations between POMCS6K1KO or AgRPS6K1KO mice and their respective controls in either gender (Figures 2E–2H, S1L, and S1M; data not shown). POMCS6K1KO mice had a modest increase in fasted serum leptin levels on a HFD at 34 weeks of age despite their normal adiposity (Figure S1L). Light- or dark-phase energy expenditure, respiratory exchange ratio, and locomotion were unchanged between chow-fed WT and POMCS6K1KO or AgRPS6K1KO mice (Table S1). These data suggest that ablation of *Rps6kb1* signaling in POMC and AgRP neurons does not affect food intake or long-term bodyweight regulation.

Glucose Homeostasis in POMCS6K1 and AgRPS6K1 Mice

As POMC and AgRP neurons regulate peripheral glucose homeostasis, we studied this in POMCS6K1KO or AgRPS6K1KO mice on chow and HFD. Fed or fasted serum glucose levels, fasted insulin levels (Table S2), and glucose tolerance were not different between control and mutant mice regardless of age (8 and 26 weeks) or diet (data not shown), while POMCS6K1KO mice had a mild impairment in insulin sensitivity on chow (area under the curve: WT, 79.5 ± 2.0 versus KO, 86.7 ± 3.1, n = 20–22, $p < 0.05$).

Hyperinsulinemic-euglycemic clamp studies were conducted to reveal any potential tissue-specific regulation of glucose homeostasis. Serum glucose concentrations, glucose infusion rate (GIR), and insulin concentrations before and at the end of the clamp were equivalent between male WT and POMCS6K1KO or AgRPS6K1KO mice (data not shown). Under basal conditions, whole-body glucose utilization, equivalent to endogenous hepatic glucose production (HGP), did not differ between WT and POMCS6K1KO or AgRPS6K1KO mice (Figures 2I and 2J). During the hyperinsulinemic clamp, suppression of HGP was impaired in POMCS6K1KO mice compared with controls

OPEN
ACCESS
CellPress

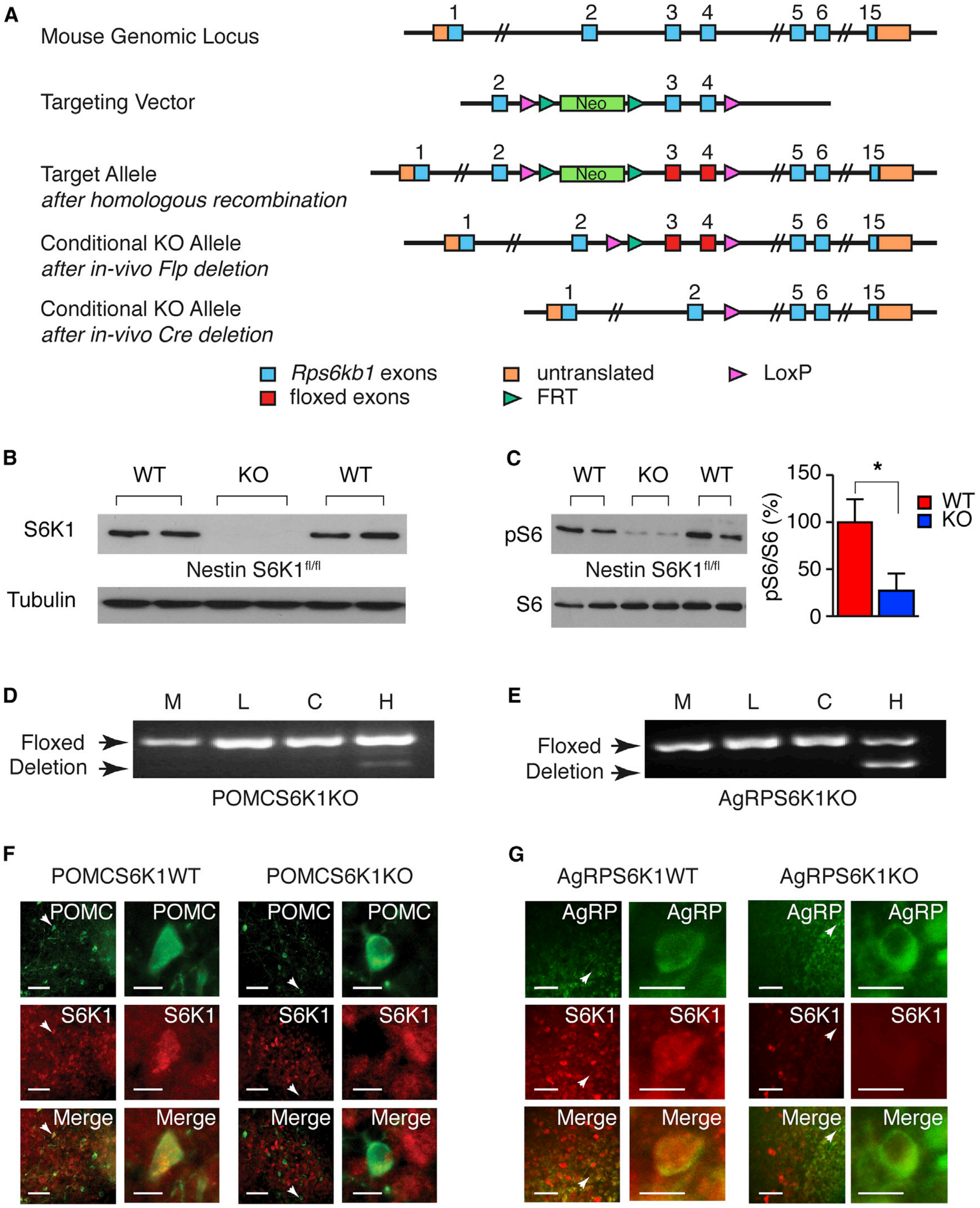

Figure 1. Generation of Conditional *Rps6kb1* Floxed Mice

(A) Schematic diagram of the targeting approach for *Rps6kb1* before and after homologous recombination into the mouse genomic locus. The neomycin (Neo) resistance gene was removed by breeding with *Flpe* mice.

(B) Representative western blot analysis of S6K1 from brain lysates of nestin-cre S6K1$^{fl/fl}$ WT and KO mice.

(C) Western blots of phosphorylated S6 (pS6, top) and total S6 (bottom) in brain lysates from nestin-cre S6K1$^{fl/fl}$ WT and KO mice. Bar chart of the ratio between pS6 and total S6 is show on the right. n = 4–5 mice per genotype, mean ± SEM, *p < 0.05.

(D and E) Representative PCR analysis for the floxed allele (top) and recombination (deletion) event (bottom) in POMCS6K1KO (D) and AgRPS6K1KO (E) mice. M, skeletal muscle; L, liver; C, cerebral cortex; H, hypothalamus.

(F and G) Fluorescent (green) arcuate POMC (F) and AgRP (G) neurons in WT (left two panels) and KO (right two panels) mice are shown adjacently at low and high magnifications. Representative S6K1 labeled neurons (red) co-localize with POMC and AgRP neurons in WT but not *Rps6kb1*-deleted cells. Scale bars represent 50 and 10 μm for low- and high-magnification images, respectively.

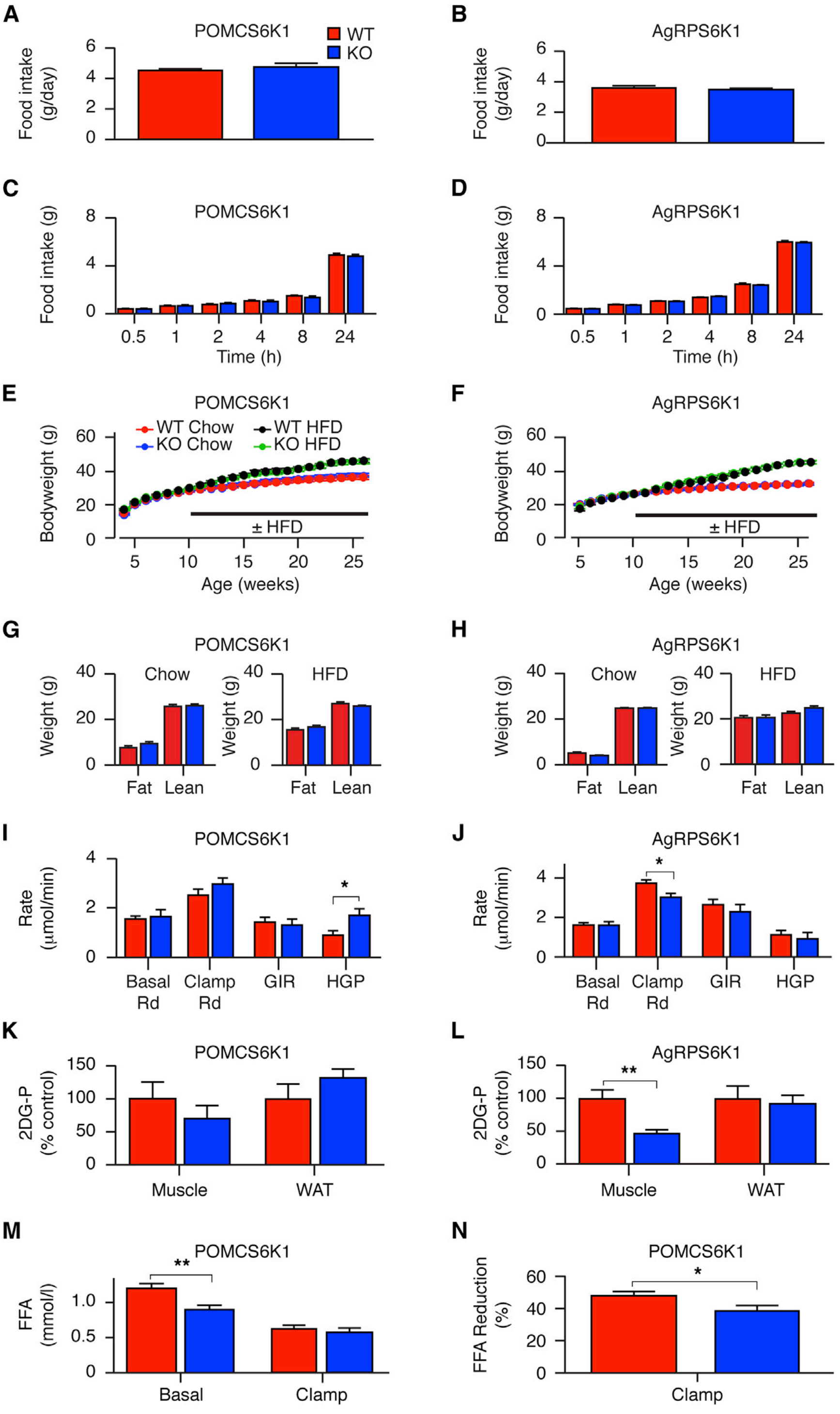

Figure 2. POMC and AgRP S6K1 Regulate Peripheral Glucose Homeostasis but Not Feeding Behavior and Bodyweight

(A and B) Ad libitum food intake in male WT (red bars) and KO (blue bars) POMCS6K1 (A) and AgRPS6K1 (B) mice on normal chow. POMCS6K1, n = 20–23, and AgRPS6K1, n = 7–10 mice per genotype. Mean ± SEM.

(C and D) Cumulative food intake from WT (red) and KO (blue) male POMCS6K1 (C) and AgRPS6K1 (D) mice following an overnight fast. POMCS6K1, n = 20–23, and AgRPS6K1, n = 7–10, mice per genotype. Mean ± SEM.

(E and F) Bodyweight curves for male POMCS6K1 (E) and AgRPS6K1 (F) mutant mice on normal chow (n = 18–22 and n = 23–33 mice per genotype, respectively) or on a HFD (n = 19–21 and n = 19–26 mice per genotype, respectively). WT mice are shown by red circles on chow and black circles on HFD, whereas KO mice on chow or HFD are shown in blue or green circles, respectively. Mean ± SEM.

(G and H) EchoMRI analysis of WT (red) and KO (blue) mice shown in (E) and (F). Fat and lean mass at 34 weeks old in POMCS6K1 (G) and AgRPS6K1 (H) mice on normal chow (left) or HFD (right). Mean ± SEM.

(I and J) Glucose disposal (Rd), GIR and HGP during basal and hyperinsulinemic-euglycemic clamp conditions from WT (red) and KO (blue) POMCS6K1 (I) and AgRPS6K1 (J) mice. n = 8–9 mice per genotype, mean ± SEM, *$p < 0.05$.

(K and L) Skeletal muscle and white adipose tissue (WAT) uptake of ^{14}C-2-deoxyglucose-phosphate (2-DG-P) from WT (red) and KO (blue) POMCS6K1 (K) and AgRPS6K1 (L) mice shown in (I) and (J). Mean ± SEM, **$p < 0.001$.

(M) Serum FFA concentration before and the during hyperinsulinemic-euglycemic clamp in WT (red) and KO (blue) POMCS6K1 mice shown in (I). Mean ± SEM, **$p < 0.001$.

(N) Percentage reduction of serum FFA concentration following hyperinsulinemic-euglycemic clamp in WT (red) and KO (blue) POMCS6K1 mice shown in (I). Mean ± SEM, *$p < 0.05$.

(Figure 2I). In contrast, in AgRPS6K1KO mice, whole-body glucose utilization during the clamp was reduced when compared with controls (Figure 2J). While there were no differences in glucose uptake between WT and POMCS6K1KO mice (Figure 2K), glucose uptake into skeletal muscle was impaired in AgRPS6K1KO mice (Figure 2L).

In POMCS6K1KO compared with control mice, fasted plasma free fatty acid (FFA) levels were lower but not suppressed by the hyperinsulinemic clamp (Figures 2M and 2N). FFA levels were not different between WT and AgRPS6K1KO mice under basal (WT, 0.87 ± 0.05 versus KO, 1.07 ± 0.05 mmol/l, n = 10–11) and clamp conditions (WT, 0.40 ± 0.05 versus KO, 042 ± 0.03 mmol/l, n = 8). Total triglyceride levels were unaltered in both lines (POMC: WT, 107.7 ± 5.7 versus KO, 101.0 ± 5.5 mg/dl, n = 13; AgRP: WT, 121.4 ± 15.3 versus KO, 124.5 ± 11.4 mg/dl, n = 8–10). Together these findings indicate that loss of *Rps6kb1* in POMC neurons impairs central regulation of HGP and also impacts upon peripheral lipid metabolism, while

loss of *Rps6kb1* in AgRP neurons impairs skeletal muscle insulin sensitivity.

Mechanisms Underlying Metabolic Phenotypes in POMCS6K1KO and AgRPS6K1KO Mice

The liver expression of phosphoenolpyruvate carboxykinase-1 (*Pck1*), glucose-6-phosphatase, and interleukin-6 (a cytokine implicated in the control of HGP) were unaltered in either POMCS6K1KO or AgRPS6K1KO mice (Figures S2A and S2B). Consistent with our finding of reduced FFA levels in POMCS6K1KO mice, we found that adipose tissue mRNA levels of hormone-sensitive lipase-1 were reduced (Figure S2C), whereas those of stearoyl-CoA desaturase-1, fatty-acid synthase, diacylglycerol o-acyltransferase-2, fatty-acid binding protein-4, perilipin-1, and *Pck1* were unaltered in both POMCS6K1KO and AgRPS6K1KO mice (Figures S2C and S2D). To study connections between the melanocortin system and peripheral metabolism, we examined sympathetic nervous system function. Urinary norepinephrine and epinephrine concentrations, core temperature, and brown adipose tissue gene expression were unaltered in both POMCS6K1KO and AgRPS6K1KO mice, suggesting no major alteration in sympathetic function (Figures S2E–S2G; data not shown). Next we examined potential hypothalamic mechanisms that might underlie the changes in peripheral metabolism in POMCS6K1KO and AgRPS6K1KO mice. Hypothalamic *Agrp*, *Npy*, *Pomc*, carboxypeptidase E, and melanocortin-4 receptor mRNA expression was unaltered in POMCS6K1KO and AgRPS6K1KO mice (Figures S2H and S2I). Phosphorylation of signal transducer and activator of transcription-3 (pSTAT3), a major mediator of leptin action, was equivalent between WT and POMCS6K1KO mice (Figures S3A–S3C). S6K1 has also been reported to phosphorylate AMP-dependent protein kinase (AMPK) on serine 485/491 to mediate leptin's effect on food intake (Dagon et al., 2012). However, we found no differences in hypothalamic AMPK serine 485 phosphorylation after leptin treatment in WT and mice lacking *Rps6kb1* in all neurons (Figure S3D).

In electrophysiology studies, leptin depolarized a sub-population of WT (9 of 16) and *Rps6kb1*-deleted (8 of 15) POMC neurons (Figures S3E, S3F, and S3J). Similar to our previous studies (Al-Qassab et al., 2009; Claret et al., 2007), insulin modestly depolarized a subpopulation of WT (6 of 12 neurons; Figures S3G and S3J) but also 4 of 12 *Rps6kb1*-deleted AgRP neurons studied (Figure S3H). However, insulin also hyperpolarized 2 of 12 *Rps6kb1*-deleted AgRP neurons; thus, the population response to insulin as a whole showed no significant change (Figures S3I and S3J). Although intracerebroventricular (i.c.v.) leucine did not alter food intake, leucine (5 mM) modestly depolarized WT POMC neurons, but equivalent effects were seen in *Rps6kb1*-deleted cells (ΔVm, WT, +3.0 ± 1.1 versus KO, +3.4 ± 1.0 mV, n = 5–6, $p < 0.05$ from control; Figures S3K and S3L). These findings suggest that S6K1 signaling in POMC neurons is not required for the electrophysiological effects of leptin and leucine and abrogation of S6K1 signaling in AgRP neurons does not impact upon the electrophysiological effects of insulin.

Rps6kb1 Deletion Reduces Neuronal Excitability, Alters AgRP Neuron Glucose Sensing and Synaptic Strength and POMC Neuron Size

During these studies, it became evident that *Rps6kb1*-deleted POMC and AgRP neurons were less excitable in 5 mM external glucose (Figures 3A–3F), equivalent to fed glucose concentrations in the brain (Routh, 2002). *Rps6kb1*-deleted POMC neurons had a more hyperpolarized resting membrane potential (Vm) and lower spike frequency than control POMC neurons while input resistance was not different (Figures 3A, 3C, and 3E). *Rps6kb1*-deleted AgRP neurons also had a lower resting spike firing frequency, but Vm and input resistance were equivalent to control neurons (Figures 3B, 3D, and 3F). In separate recordings, we examined the excitable properties of POMC and AgRP neurons in 1 mM external glucose, which is similar to fasted glucose concentrations measured in the brain (Routh, 2002). Control POMC neurons had a lower Vm and input resistance when compared with recordings obtained in 5 mM glucose, yet spike frequency was not different (Figures 3A, 3C, and 3E). Input resistance in 1 mM glucose was also lower in *Rps6kb1*-deleted POMC neurons when compared with recordings at the higher glucose concentration but resting Vm and spike firing frequency were equivalent (Figures 3A, 3C, and 3E). In control AgRP neurons, lowering external glucose decreased input resistance and spike frequency when compared with recordings in 5 mM glucose, but Vm was not significantly different (Figures 3B, 3D, and 3F). However, in *Rps6kb1*-deleted AgRP neurons, Vm, spike frequency, and input resistance were equivalent between the two external glucose concentrations (Figures 3B, 3D, and 3F). Therefore, POMC neurons lacking *Rps6kb1* displayed a hyperpolarized resting membrane potential in high glucose while the biophysical properties of AgRP neurons lacking *Rps6kb1* did not alter in different glucose concentrations possibly indicating defective glucose sensing. These defects may be due to altered ATP-sensitive K^+ (K_{ATP}) channel subunit expression, and we found that *Kcjn11* expression was significantly increased in POMCS6K1KO and reduced in AgRPS6K1KO when compared with WT mice; however, *Abcc9* expression was unchanged (Figures 3G and 3H). Hypothalamic expression of glucokinase was reduced in AgRPS6K1KO but unchanged in the POMCS6K1KO mice (Figures 3G and 3H).

Changes in synaptic weight in *Rps6kb1*-deleted AgRP neurons could explain a lower firing frequency without an observable difference in resting Vm. Therefore, we recorded from WT and AgRPS6K1KO neurons and pharmacologically isolated miniature excitatory (mEPSC) and inhibitory (mIPSC) post-synaptic currents in the presence of tetrodotoxin (1 μM; Figures 4A–4H). Mean mEPSC amplitude was slightly higher in *Rps6kb1*-deleted AgRP neurons when compared with WT neurons (WT, −27.2 ± 0.2 versus KO, −29.4 ± 0.2 pA, 4,243–4,505 events from 13 neurons per genotype, $p < 0.0001$; Figures 4A and 4C). There were no differences in decay time constants or event frequency (Figures 4E and 4G) between WT and KOs. In contrast, mIPSC amplitude was significantly smaller in AgRPS6K1KO neurons when compared with control cells (WT, −81.6 ± 2.1 versus KO, −49.0 ± 0.9 pA, 898–945 events from ten neurons per genotype, $p < 0.0001$; Figures 4B and 4D). Likewise, mIPSC decay

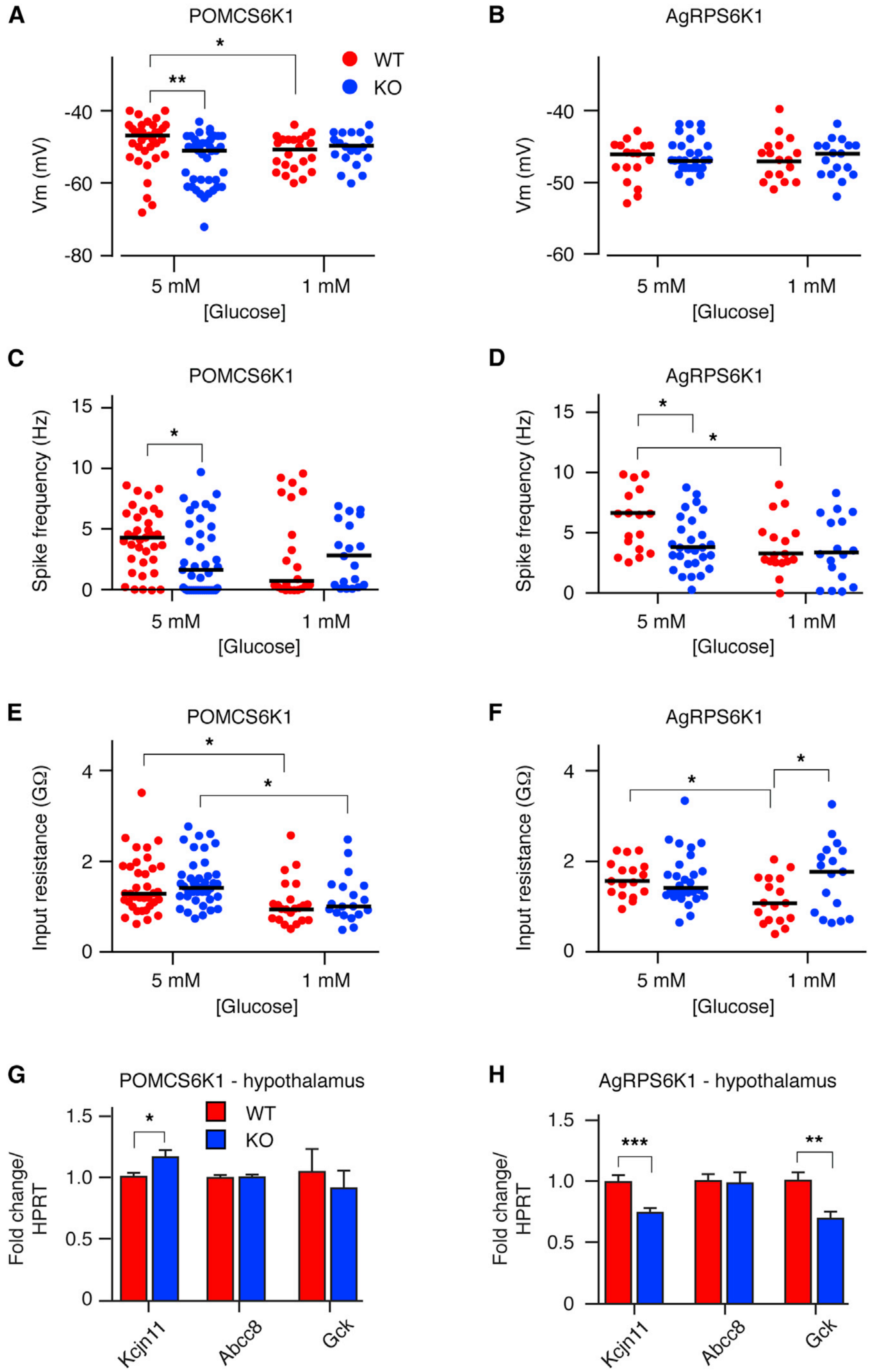

Figure 3. S6K1 Regulates the Excitable Properties of POMC and AgRP Neurons

(A–F) Scatter plots of resting membrane potentials (Vm, A and B), spike firing frequencies (C and D), and input resistances (E and F) in POMC (A, C, and E) and AgRP (B, D, and F) neurons in 5 mM (POMC, n = 36–40 and AgRP, n = 17–28 neurons per genotype) or 1 mM (POMC, n = 19–22 and AgRP, n = 17 neurons per genotype) external glucose, as indicated. Solid horizontal bars denote median values. *$p < 0.05$, **$p < 0.001$. WT and KO neurons are shown in red and blue circles, respectively.

(G and H) Quantitative PCR for Kir6.2 (*Kcjn11*), SUR1 (*Abcc8*) and glucokinase (*Gck*) from the basomedial hypothalamus of fasted POMCS6K1 (G) and AgRPS6K1 (H) mice. n = 10–12 mice per genotype. Mean ± SEM, *$p < 0.05$, **$p < 0.001$, ***$p < 0.0001$. WT and KO neurons are shown in red and blue bars, respectively.

constants were slower in *Rps6kb1*-deleted neurons when compared with WT controls (WT, 9.9 ± 0.1 versus KO, 10.7 ± 0.2 ms, 898–945 events from ten neurons per genotype, $p < 0.001$; Figure 4F). Spontaneous mIPSC frequency was the same between WT and *Rps6kb1*-deleted AgRP neurons (Figure 4H). Differences in the expression of $GABA_A$ receptor alpha subunits may lead to alterations in kinetics, but we found no changes in the expression of $GABA_A$ receptor alpha-subunits 1, 2, 3 and 5 in the hypothalamus of AgRPS6K1KO mice (Figure 4I). Together, these data show that chronic deletion of *Rps6kb1* in AgRP neurons alters synaptic strength.

Next, we examined neuronal architecture as S6K1 has been implicated in cell growth. Initial indirect measurement of cell size through cell capacitance measurements did not identify a difference between control and *Rps6kb1*-deleted POMC and AgRP neurons (Figure S4A). However, in immunohistochemistry studies, somatic diameter and area was approximately 5% smaller in *Rps6kb1*-KO when compared with WT POMC neurons (Figures S4B–S4D), while no overt differences in POMC fibers in areas such as the hypothalamic paraventricular nucleus were observed (data not shown).

DISCUSSION

Hypothalamic S6K1 has been implicated in the regulation of food intake, energy expenditure, and bodyweight (Blouet and Schwartz, 2010; Haissaguerre et al., 2014). In contrast, in our studies specifically ablating *Rps6kb1* in POMC or AgRP neurons, we find that this signaling pathway does not play an indispensable role in regulating these processes. We instead reveal a key role for both POMC and AgRP neuronal S6K1 in peripheral glucose metabolism and show that deletion of *Rps6kb1* alters POMC and AgRP neuron excitability and disrupts glucose sensing in AgRP neurons. Potential explanations for the differences between our findings and previous work include the short-time window but high level of adenovirally mediated gene expression, the lack of cell-selective manipulations, the presence of local hypothalamic inflammation, acute post-operative weight loss, and

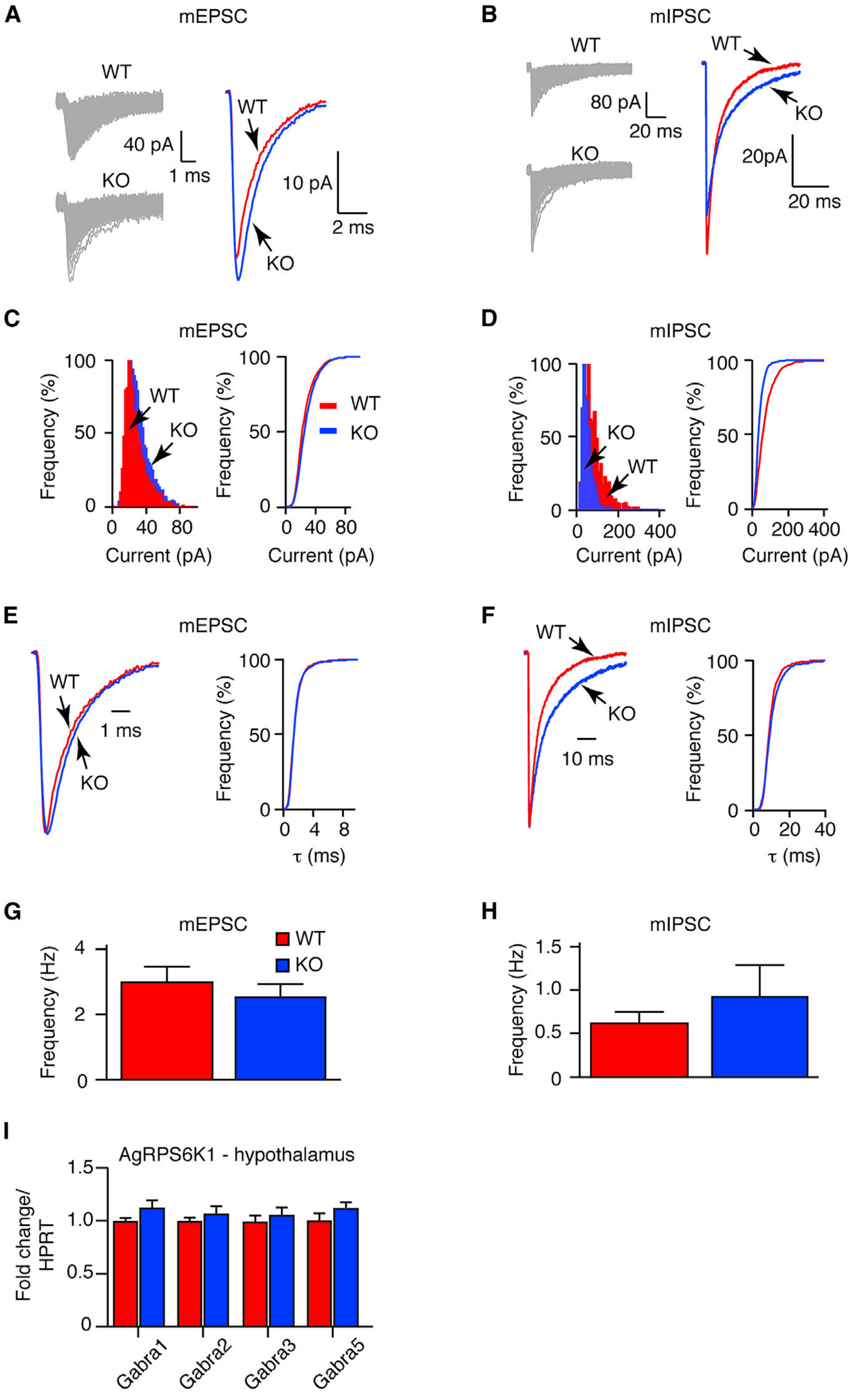

Figure 4. Synaptic Strength in AgRP Neurons Is Altered by *Rps6kb1* Deletion

(A and B) Representative overlapping (left) and composite (right) miniature excitatory (A) and inhibitory (B) post-synaptic currents (mEPSC and mIPSC, respectively) in WT and *Rps6kb1*-deleted (KO) AgRP neurons.

(C and D) Frequency histograms (left) and cumulative frequency curves (right) are shown for mEPSC (C) and mIPSC (D) amplitudes in WT (red) and KO (blue) AgRP neurons. n = 10–13 neurons per genotype.

(E and F) Normalized composite traces (left) with cumulative frequency curves (right) for decay times of mEPSC (E) and mIPSC (F) in WT (red) and KO (blue) AgRP neurons. n = 10–13 neurons per genotype.

(G and H) Bar charts of spontaneous mEPSC (G) and mIPSC (H) event frequency in WT (red) and KO (blue) AgRP neurons. n = 10–13 neurons per genotype. Mean ± SEM.

(I) Quantitative PCR analysis for $GABA_A$ receptor alpha subunits 1, 2, 3, and 5 (*Gabra1*, *Gabra2*, *Gabra3*, *Gabra5*, respectively) in the MBH of fasted WT (red) and KO (blue) AgRPS6K1 mice. n = 10–12 mice per genotype. Mean ± SEM.

anorexia complicating the interpretation of feeding and bodyweight data and potential off-target effects of the constructs. Chronic deletion may also lead to compensatory changes such as upregulation of S6K2, but in our preliminary studies deleting both *Rps6kb1* and *Rps6kb2* in POMC neurons, we find no changes in food intake or bodyweight (Figures S4E and S4F). It is also conceivable that the effects of S6K1 on energy balance are due to signaling in other MBH cell populations. Yet, this is unlikely as global neuronal deletion of *Rps6kb1* achieved using nestin-cre mice did not lead to alterations in bodyweight or insulin tolerance (Figures S4G and S4H). In contrast, our broad range of relevant physiological, molecular, and electrophysiological data indicate that S6K1 is not required for the hypothalamic regulation of food intake, bodyweight, and energy expenditure.

Rapamycin blocks the anorexigenic effects of leptin (Cota et al., 2006), but the lack of effect of *Rps6kb1*-deletion on acute leptin regulation of both food intake and POMC neuron excitability and signaling suggests the involvement of an alternative rapamycin-sensitive mechanism. S6K1 also phosphorylates AMPK on serine 485/491, an event that inhibits AMPK activity and is required for leptin's effect on food intake (Dagon et al., 2012). However, we were unable to detect differences in AMPK serine 485 phosphorylation after leptin treatment between WT and mice lacking *Rps6kb1* in all neurons. Ghrelin action in AgRPS6K1KO mice was also normal. S6K1 has also been suggested to mediate the anorexigenic effects of central administration of leucine (Blouet et al., 2009; Cota et al., 2008), and while we were unable to detect a suppression of food intake, the persistence of leucine's electrophysiological actions in *Rps6kb1*-deficient POMC

neurons suggest that S6K1 is not required for this component of leucine's actions.

We find that S6K1 signaling in POMC and AgRP neurons regulates glucose homeostasis. Previous studies have given conflicting results, with one suggesting that hypothalamic activation of S6K1 signaling causes hepatic insulin resistance and its inhibition improves hepatic insulin sensitivity (Blouet et al., 2008), while another showed essentially the opposite findings (Ono et al., 2008). We show that deletion of *Rps6kb1* in POMC neurons leads to defective regulation of HGP, while deletion in AgRP neurons causes skeletal muscle insulin resistance. In POMC neurons leptin receptor signaling alone, or in combination with insulin receptor signaling, and phosphatidylinositol-3 kinase signaling have both been shown to regulate HGP (Berglund et al., 2012; Hill et al., 2010; Hill et al., 2008). Insulin-receptor signaling in AgRP neurons likewise regulates HGP (Könner et al., 2007). Melanocortin circuits control both HGP and insulin sensitivity in skeletal muscle and adipose tissue function (Berglund et al., 2014; Nogueiras et al., 2007; Obici et al., 2001; Rossi et al., 2011). Our studies implicate S6K1 signaling in POMC neurons as one of the mechanisms by which this cell type regulates HGP and peripheral lipid metabolism. We also show a role for AgRP neurons in regulating skeletal muscle sensitivity and implicate S6K1 signaling in this process.

Hypothalamic neuronal excitability is modulated by external glucose by inhibition of K_{ATP} channels following the uptake and metabolism of glucose (Levin et al., 2004). Glucose sensing in POMC neurons has been shown to regulate peripheral glucose homeostasis (Parton et al., 2007), and reducing glucose to hypoglycemic conditions hyperpolarizes both POMC and AgRP neurons (Claret et al., 2007). Consistent with the opening of a resting conductance, input resistance in both POMC and AgRP neuronal populations was lower in an external glucose concentration (1 mM) reflecting the fasted state (Routh, 2002) in comparison with recordings obtained in a higher glucose concentration (5 mM). However, low glucose did not alter the biophysics of AgRPS6K1KO neurons, which may indicate a loss of glucose sensing in these cells, which could underpin alterations in peripheral glucose metabolism. A reduction in the expression of a pore-forming subunit of K_{ATP} channels and the enzyme glucokinase, which initiates glucose metabolism may contribute to this loss of glucose sensing. POMCS6K1KO neurons had a lower input resistance when compared with recordings in higher glucose concentrations, suggesting that glucose sensing is intact. However, these neurons had lower resting membrane potential and spike firing frequency than control POMC neurons, which may imply a reduction in basal transmitter release, which could alter peripheral glucose homeostasis. The properties and kinetics of voltage-dependent conductances or the electrogenic properties of the neuron due to changes in anatomy may be modulated by S6K1 to alter resting excitability. Although cell capacitance measurements in *Rps6kb1*-deficient and control POMC neurons did not suggest alterations, in immunohistochemical anatomical assessments, we found a very small reduction in soma size. One caveat with electrophysiological recordings of individual neurons is that this technique can only capture small samples within the wider neuronal population. In the case of POMC and AgRP neurons, which display some functional heterogeneity and do not all respond to the same hormones and nutrients, it is possible that some neurons that have altered responses are undetected.

In summary, our studies define the physiological and cellular roles of S6K1 signaling in POMC and AgRP neurons and give insights into the role of this pathway in the CNS regulation of energy homeostasis. We find no role for hypothalamic S6K1 in the regulation of feeding and bodyweight but demonstrate a key role for this molecule in glucose homeostasis.

EXPERIMENTAL PROCEDURES

Mice and Animal Care

The use and genotyping of POMCCre, AgRPCre (both on a C57Bl/6 background), and NestinCre (on a mixed C57Bl/6 × 129sv background) mice have been previously described (Al-Qassab et al., 2009; Choudhury et al., 2005; Claret et al., 2011). Mice with floxed alleles for *Rps6kb1* on a C57Bl/6 background were generated by Taconic Biosciences and crossed with the *Cre*-expressing transgenic mice to generate compound heterozygotes. These mice were intercrossed with $Rps6kb1^{fl/wt}$ mice to obtain WT ($Cre^{-/-}/Rps6kb1^{wt/wt}$, $Cre^{-/-}/Rps6kb1^{fl/fl}$, $Cre^{+/-}/Rps6kb1^{wt/wt}$), and KO ($Cre^{+/-}/Rps6kb1^{fl/fl}$) mice for each line. To generate mice lacking floxed alleles but expressing GFP or YFP in cells harboring the deletion event, mice were bred with POMC-GFP or Rosa26YFP indicator mice and bred to homozygosity for the floxed allele. Mice were maintained on a 12-hr light/dark cycle with free access to water and standard mouse chow (4.25% fat, RM3; Special Diet Services) or HFD (45% fat, D12451; Research Diets) and housed in specific-pathogen free barrier facilities in individually ventilated cages of mixed genotypes. All KO and transgenic mice were studied with appropriate littermates of the three control genotypes. Mice were handled and all in vivo studies performed in accordance to the United Kingdom Animals (Scientific Procedures) Act (1986) and approved by Imperial College's Animal Welfare and Ethical Review Body.

Statistical Analysis

Data are expressed as mean ± SEM unless otherwise stated, and the single animal was the unit of analysis unless otherwise stated. Statistical significance was calculated at the 95% level of confidence using parametric (unpaired and paired t tests, one-way ANOVA, repeated-measures two-way ANOVA) or non-parametric (Kruskal-Wallis) tests with post hoc Bonferroni's or Dunn's multiple comparison analysis, where appropriate. Statistical significance was calculated from all electrophysiological recordings (responsive and non-responsive).

SUPPLEMENTAL INFORMATION

Supplemental Information includes Supplemental Experimental Procedures, four figures, and two tables and can be found with this article online at http://dx.doi.org/10.1016/j.celrep.2015.03.029.

AUTHOR CONTRIBUTIONS

M.A.S., L.K., E.E.I., M.K.H., S.M.A.P., and M.W.G. generated and phenotyped the mouse lines. M.A.S. and E.E.I. performed i.c.v. feeding studies. M.A.S. performed and analyzed the electrophysiological studies. L.K., M.K.H., S.M.A.P., and A.I.C. performed and analyzed PCR analysis. L.K., A.W., and D.C. undertook and conceived western blot analysis. P.J.V. and A.V.-P. designed and conducted hyperinsulinemic-euglycemic clamp studies. M.A.S. and D.J.W. conceived and designed the study and wrote the manuscript. All authors contributed to the editing of the manuscript.

ACKNOWLEDGMENTS

We thank Olga Boruc for technical assistance and Taconic Biosciences for generation of mice with a floxed allele of S6K1. Work in the Smith lab was funded by Diabetes UK (12/0004486) and Biotechnology and Biological

Sciences Research Council (BB/I00842X/1). Work in the Withers (MC-A654-5QB40), Carling (MC-A654-5QB10), Voshol, and Vidal-Puig (MRC_MC_UU_12012/5) labs was funded by the Medical Research Council. D.J.W. also received funding through a Wellcome Trust Strategic Award (098565).

Received: December 18, 2014
Revised: February 11, 2015
Accepted: March 11, 2015
Published: April 9, 2015

REFERENCES

Al-Qassab, H., Smith, M.A., Irvine, E.E., Guillermet-Guibert, J., Claret, M., Choudhury, A.I., Selman, C., Piipari, K., Clements, M., Lingard, S., et al. (2009). Dominant role of the p110beta isoform of PI3K over p110alpha in energy homeostasis regulation by POMC and AgRP neurons. Cell Metab. *10*, 343–354.

André, C., and Cota, D. (2012). Coupling nutrient sensing to metabolic homoeostasis: the role of the mammalian target of rapamycin complex 1 pathway. Proc. Nutr. Soc. *71*, 502–510.

Berglund, E.D., Vianna, C.R., Donato, J., Jr., Kim, M.H., Chuang, J.C., Lee, C.E., Lauzon, D.A., Lin, P., Brule, L.J., Scott, M.M., et al. (2012). Direct leptin action on POMC neurons regulates glucose homeostasis and hepatic insulin sensitivity in mice. J. Clin. Invest. *122*, 1000–1009.

Berglund, E.D., Liu, T., Kong, X., Sohn, J.W., Vong, L., Deng, Z., Lee, C.E., Lee, S., Williams, K.W., Olson, D.P., et al. (2014). Melanocortin 4 receptors in autonomic neurons regulate thermogenesis and glycemia. Nat. Neurosci. *17*, 911–913.

Blouet, C., and Schwartz, G.J. (2010). Hypothalamic nutrient sensing in the control of energy homeostasis. Behav. Brain Res. *209*, 1–12.

Blouet, C., Ono, H., and Schwartz, G.J. (2008). Mediobasal hypothalamic p70 S6 kinase 1 modulates the control of energy homeostasis. Cell Metab. *8*, 459–467.

Blouet, C., Jo, Y.H., Li, X., and Schwartz, G.J. (2009). Mediobasal hypothalamic leucine sensing regulates food intake through activation of a hypothalamus-brainstem circuit. J. Neurosci. *29*, 8302–8311.

Choudhury, A.I., Heffron, H., Smith, M.A., Al-Qassab, H., Xu, A.W., Selman, C., Simmgen, M., Clements, M., Claret, M., Maccoll, G., et al. (2005). The role of insulin receptor substrate 2 in hypothalamic and beta cell function. J. Clin. Invest. *115*, 940–950.

Claret, M., Smith, M.A., Batterham, R.L., Selman, C., Choudhury, A.I., Fryer, L.G., Clements, M., Al-Qassab, H., Heffron, H., Xu, A.W., et al. (2007). AMPK is essential for energy homeostasis regulation and glucose sensing by POMC and AgRP neurons. J. Clin. Invest. *117*, 2325–2336.

Claret, M., Smith, M.A., Knauf, C., Al-Qassab, H., Woods, A., Heslegrave, A., Piipari, K., Emmanuel, J.J., Colom, A., Valet, P., et al. (2011). Deletion of Lkb1 in pro-opiomelanocortin neurons impairs peripheral glucose homeostasis in mice. Diabetes *60*, 735–745.

Cota, D., Proulx, K., Smith, K.A., Kozma, S.C., Thomas, G., Woods, S.C., and Seeley, R.J. (2006). Hypothalamic mTOR signaling regulates food intake. Science *312*, 927–930.

Cota, D., Matter, E.K., Woods, S.C., and Seeley, R.J. (2008). The role of hypothalamic mammalian target of rapamycin complex 1 signaling in diet-induced obesity. J. Neurosci. *28*, 7202–7208.

Dagon, Y., Hur, E., Zheng, B., Wellenstein, K., Cantley, L.C., and Kahn, B.B. (2012). p70S6 kinase phosphorylates AMPK on serine 491 to mediate leptin's effect on food intake. Cell Metab. *16*, 104–112.

Haissaguerre, M., Saucisse, N., and Cota, D. (2014). Influence of mTOR in energy and metabolic homeostasis. Mol. Cell. Endocrinol. *397*, 67–77.

Hill, J.W., Williams, K.W., Ye, C., Luo, J., Balthasar, N., Coppari, R., Cowley, M.A., Cantley, L.C., Lowell, B.B., and Elmquist, J.K. (2008). Acute effects of leptin require PI3K signaling in hypothalamic proopiomelanocortin neurons in mice. J. Clin. Invest. *118*, 1796–1805.

Hill, J.W., Elias, C.F., Fukuda, M., Williams, K.W., Berglund, E.D., Holland, W.L., Cho, Y.R., Chuang, J.C., Xu, Y., Choi, M., et al. (2010). Direct insulin and leptin action on pro-opiomelanocortin neurons is required for normal glucose homeostasis and fertility. Cell Metab. *11*, 286–297.

Könner, A.C., Janoschek, R., Plum, L., Jordan, S.D., Rother, E., Ma, X., Xu, C., Enriori, P., Hampel, B., Barsh, G.S., et al. (2007). Insulin action in AgRP-expressing neurons is required for suppression of hepatic glucose production. Cell Metab. *5*, 438–449.

Kopelman, P.G. (2000). Obesity as a medical problem. Nature *404*, 635–643.

Laplante, M., and Sabatini, D.M. (2012). mTOR signaling in growth control and disease. Cell *149*, 274–293.

Levin, B.E., Routh, V.H., Kang, L., Sanders, N.M., and Dunn-Meynell, A.A. (2004). Neuronal glucosensing: what do we know after 50 years? Diabetes *53*, 2521–2528.

Magnuson, B., Ekim, B., and Fingar, D.C. (2012). Regulation and function of ribosomal protein S6 kinase (S6K) within mTOR signalling networks. Biochem. J. *441*, 1–21.

Martins, L., Fernández-Mallo, D., Novelle, M.G., Vázquez, M.J., Tena-Sempere, M., Nogueiras, R., López, M., and Diéguez, C. (2012). Hypothalamic mTOR signaling mediates the orexigenic action of ghrelin. PLoS ONE *7*, e46923.

Morton, G.J., Meek, T.H., and Schwartz, M.W. (2014). Neurobiology of food intake in health and disease. Nat. Rev. Neurosci. *15*, 367–378.

Nogueiras, R., Wiedmer, P., Perez-Tilve, D., Veyrat-Durebex, C., Keogh, J.M., Sutton, G.M., Pfluger, P.T., Castaneda, T.R., Neschen, S., Hofmann, S.M., et al. (2007). The central melanocortin system directly controls peripheral lipid metabolism. J. Clin. Invest. *117*, 3475–3488.

Obici, S., Feng, Z., Tan, J., Liu, L., Karkanias, G., and Rossetti, L. (2001). Central melanocortin receptors regulate insulin action. J. Clin. Invest. *108*, 1079–1085.

Ono, H., Pocai, A., Wang, Y., Sakoda, H., Asano, T., Backer, J.M., Schwartz, G.J., and Rossetti, L. (2008). Activation of hypothalamic S6 kinase mediates diet-induced hepatic insulin resistance in rats. J. Clin. Invest. *118*, 2959–2968.

Parton, L.E., Ye, C.P., Coppari, R., Enriori, P.J., Choi, B., Zhang, C.Y., Xu, C., Vianna, C.R., Balthasar, N., Lee, C.E., et al. (2007). Glucose sensing by POMC neurons regulates glucose homeostasis and is impaired in obesity. Nature *449*, 228–232.

Rossi, J., Balthasar, N., Olson, D., Scott, M., Berglund, E., Lee, C.E., Choi, M.J., Lauzon, D., Lowell, B.B., and Elmquist, J.K. (2011). Melanocortin-4 receptors expressed by cholinergic neurons regulate energy balance and glucose homeostasis. Cell Metab. *13*, 195–204.

Routh, V.H. (2002). Glucose-sensing neurons: are they physiologically relevant? Physiol. Behav. *76*, 403–413.

Varela, L., and Horvath, T.L. (2012). Leptin and insulin pathways in POMC and AgRP neurons that modulate energy balance and glucose homeostasis. EMBO Rep. *13*, 1079–1086.

Williams, K.W., and Elmquist, J.K. (2012). From neuroanatomy to behavior: central integration of peripheral signals regulating feeding behavior. Nat. Neurosci. *15*, 1350–1355.

Zhang, H., Zhang, G., Gonzalez, F.J., Park, S.M., and Cai, D. (2011). Hypoxia-inducible factor directs POMC gene to mediate hypothalamic glucose sensing and energy balance regulation. PLoS Biol. *9*, e1001112.

Cell Reports
Supplemental Information

Ribosomal S6K1 in POMC and AgRP Neurons Regulates Glucose Homeostasis but Not Feeding Behavior in Mice

Mark A. Smith, Loukia Katsouri, Elaine E. Irvine, Mohammed K. Hankir, Silvia M.A. Pedroni, Peter J. Voshol, Matthew W. Gordon, Agharul I. Choudhury, Angela Woods, Antonio Vidal-Puig, David Carling, and Dominic J. Withers

Figure S1. Related to Figure 2

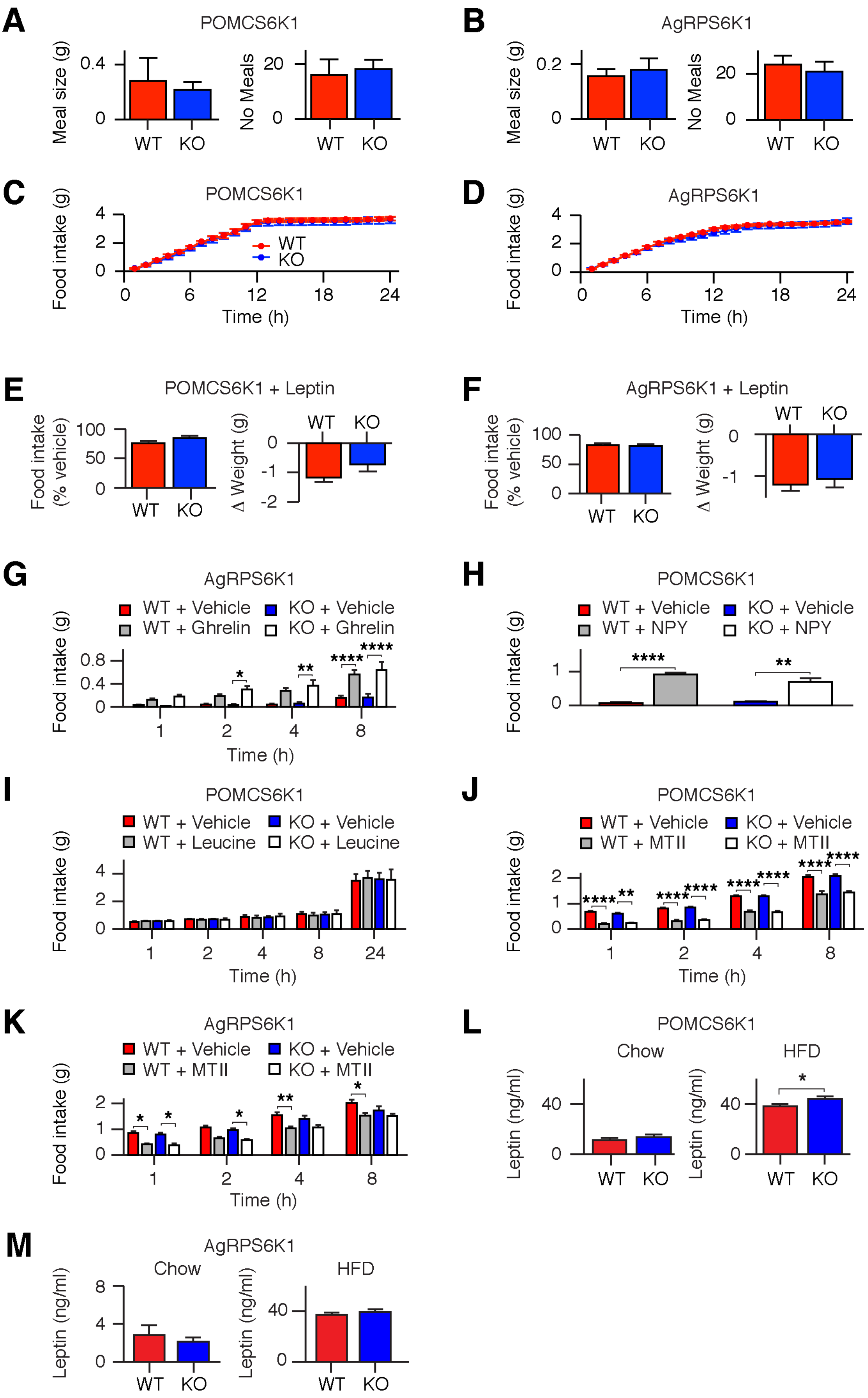

Figure S1. S6K1 is not required for the regulation of food intake

(A-D) Automated food intake monitoring of meal size (A and B, left), meal number (A and B, right) and cumulative food intake (C and D) for ad-libitum fed wild-type (WT, red bars/circles) and knockout (KO, blue bars/circles) male POMCS6K1 (A and C) and female AgRPS6K1 (B and D) mice. N=6-7 mice per genotype. Data presented is mean ± SEM.

(E and F) Leptin-induced (i.p. 0.3 mg/kg twice daily) reduction in food intake (left) and bodyweight (right) over 3 days in male POMCS6K1 (E) and AgRPS6K1 (F) WT (red bar) and KO (blue bar) mice. POMC: N=17-21; AgRP: N=17-19 mice per genotype. Data presented is mean ± SEM.

(G) Cumulative food intake for WT and KO AgRPS6K1 mice treated with vehicle (red and blue bars, respectively) or ghrelin (i.p. 5 mg/kg) (grey and white bars, respectively). N=10-12 mice per genotype. Data presented is mean ± SEM. *P<0.05, **P<0.001, ****P<0.00001.

(H) One hour food intake in WT and KO POMCS6K1 mice i.c.v. injected with vehicle (red and blue bars, respectively) or NPY (1 μg) (grey and white bars, respectively). N=3-8 mice per genotype or treatment. Data presented is mean ± SEM. **P<0.001, ****P<0.00001.

(I) Cumulative food intake in overnight fasted WT and KO POMCS6K1 mice i.c.v. injected with vehicle (red and blue bars, respectively) or leucine (2.2 μg) (grey and white bars, respectively). N=9-12 mice per genotype. Data presented is mean ± SEM.

(J and K) Cumulative food intake for WT and KO POMCS6K1 (J) and AgRPS6K1 (K) mice treated with vehicle (red and blue bars, respectively) or melanotan-II (MTII) (i.p. 2 mg/kg) (grey and white bars, respectively). N=9-12 and N=6 mice per genotype for POMCS6K1 and AgRPS6K1, respectively. Data presented is mean ± SEM. *P<0.05, **P<0.001, ****P<0.00001.

(L and M) Fasted serum leptin concentrations for WT (red bars) and KO (blue bars) mice shown in Figure 2 panels E and F. Data shown is from 34 week old POMCS6K1 (L) and AgRPS6K1 (M) mice fed on normal chow (left) or HFD (right) and shows mean ± SEM. *P<0.05.

Figure S2. Related to Figure 2

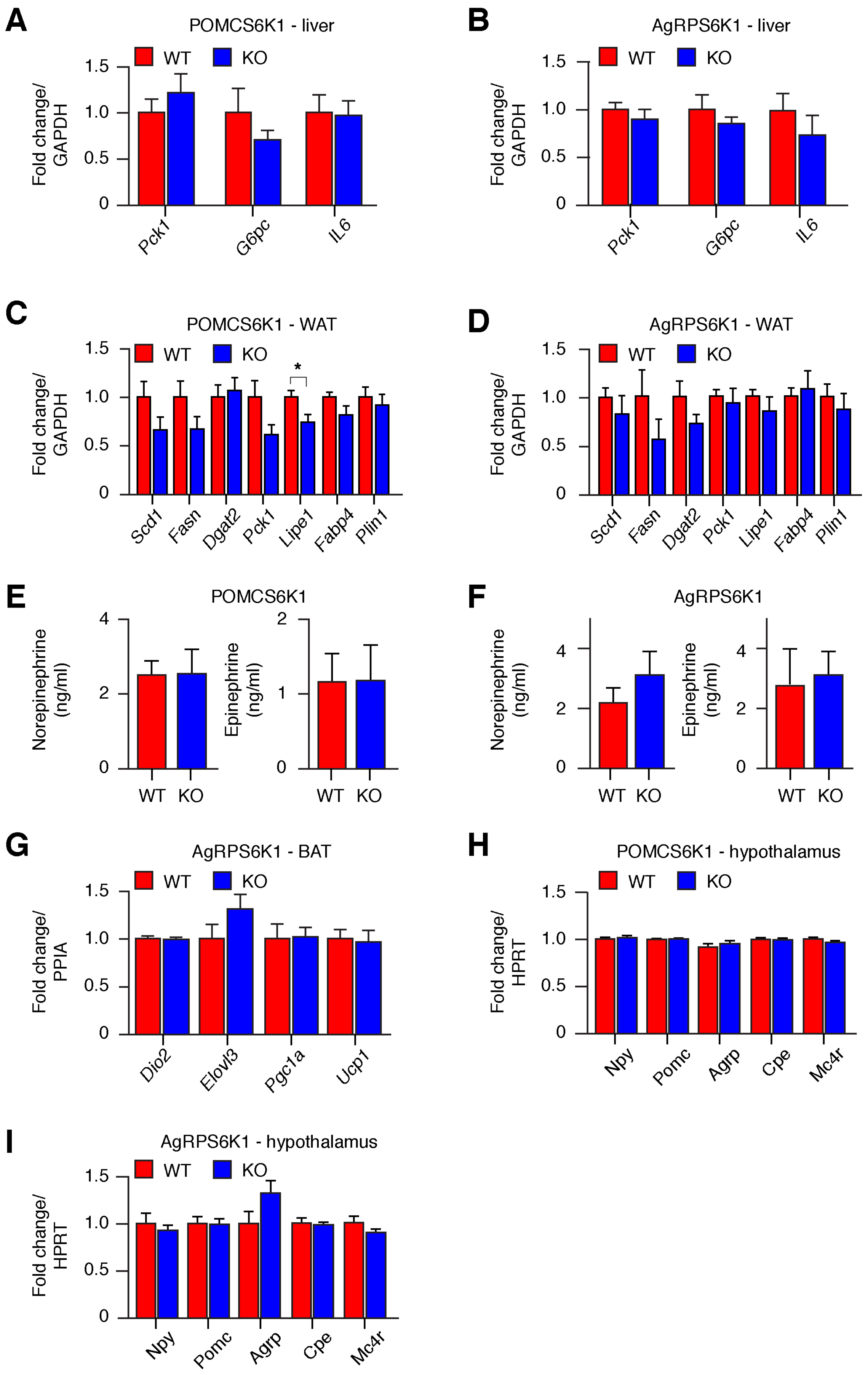

Figure S2. Gene expression in peripheral and hypothalamic tissues and urinary catecholamine concentrations

(A and B) Expression of mRNA for phosphoenolpyruvate carboxykinase-1 (*Pck1*), glucose-6-phosphatase (*G6pc*) and interleukin-6 (*IL6*) in liver of fasted wild-type (WT, red bar) and knockout (KO, blue bar) POMCS6K1 (A) or AgRPS6K1 mice (B). N=6-12 mice per genotype. Data presented is mean ± SEM.

(C and D) Expression of mRNA for stearoyl-CoA desaturase-1 (*Scd1*), fatty-acid synthase (*Fasn*), diacylglycerol o-acyltransferase 2 (*Dgat2*), *Pck1*, hormone-sensitive lipase-1 (*Lipe*), fatty-acid binding protein-4 (*Fabp4*) and perilipin-1 (*Plin1*) in white adipose tissue (WAT) of fasted WT (red bar) and KO (blue bar) POMCS6K1 (C) and AgRPS6K1 (D) mice. N=6-10 mice per genotype. Data presented is mean ± SEM. *P<0.05.

(E and F) Urinary concentrations of norepinephrine (left, N=7-13 mice per genotype) and epinephrine (right, N=4-7 mice per genotype) in fed WT (red bar) and KO (blue bar) POMCS6K1 (E) and AgRPS6K1 (F) mice. Data presented is mean ± SEM.

(G) Expression of mRNA for type-II iodothyronine deiodinase (*Dio2*), elongation of very long chain fatty acids-3 (*Elovl3*), peroxisome proliferator-activator receptor-γ coactivator-1α (*Pgc1a*) and uncoupling protein-1 (*Ucp1*) in brown adipose tissue (BAT) of fasted WT (red bar) and KO (blue bar) AgRPS6K1 mutant mice. N=9-10 mice per genotype. Data presented is mean ± SEM.

(H and I) Expression of mRNA for neuropeptide Y (*Npy*), pro-opiomelanocortin (*Pomc*), agouti-related peptide (*Agrp*), carboxypeptidase E (*Cpe*) and melanocortin-4 receptor (*Mc4r*) in the mediobasal hypothalamus of fasted WT (red bars) and KO (blue bars) POMCS6K1 (H, N=18-21 mice per genotype) or AgRPS6K1 (I, N=12 mice per genotype) mice. Data presented is mean ± SEM.

Figure S3. Related to Figure 2

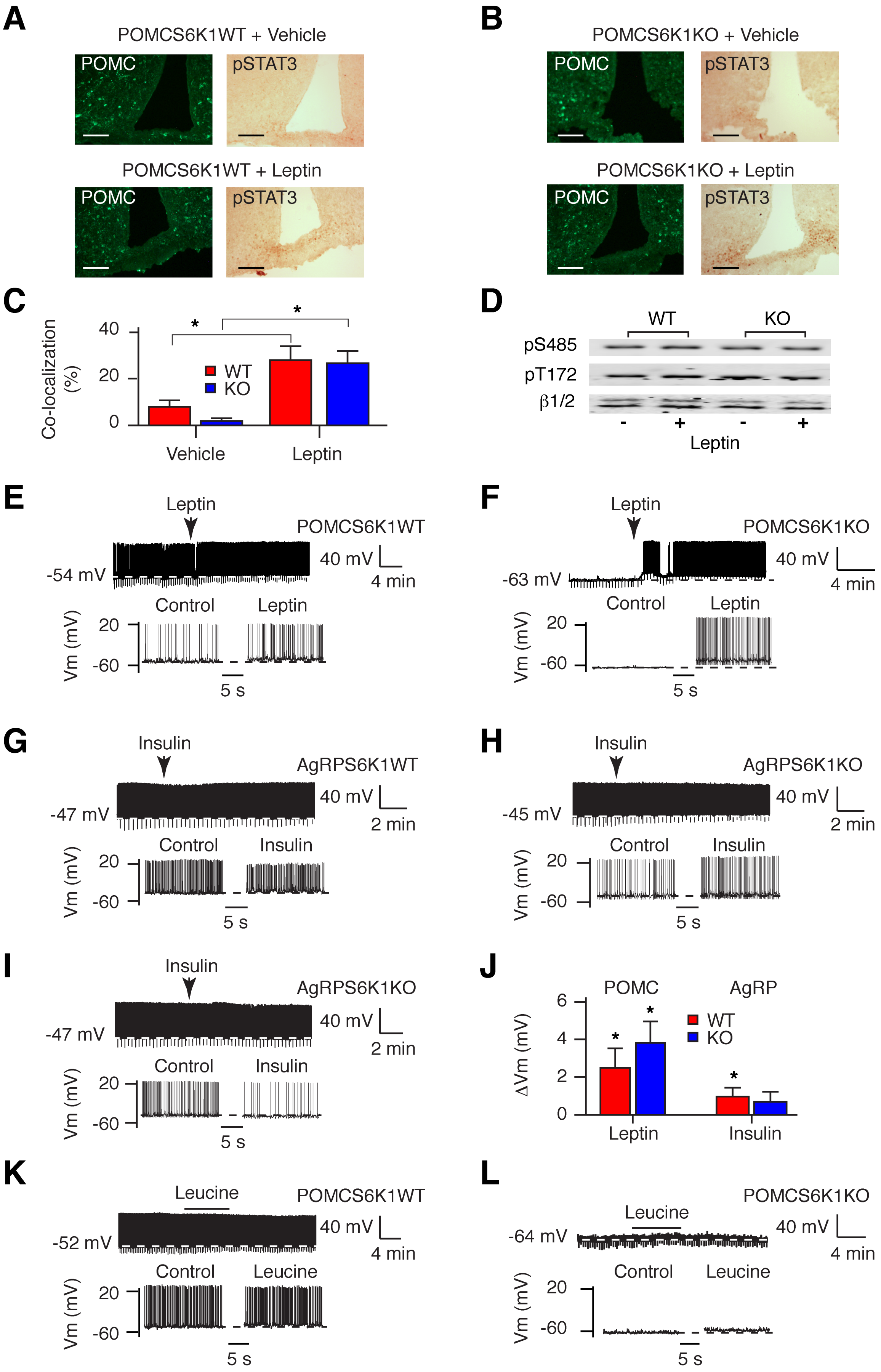

Figure S3. Leptin sensitivity is not affected by *Rps6kb1* deletion

(A and B) Representative hypothalamic sections showing immunohistochemistry for POMC (left) and phosphorylation of STAT3 (pSTAT3) (right) in POMCS6K1 wild-type (WT, A) and knockout mice (KO, B) treated with vehicle (top) or i.p. 5 mg/kg leptin (bottom). Scale bar represents 100 μm.

(C) Quantification of co-localization between POMC and pSTAT3 positive neurons. N=3-5 mice per genotype and treatment. Data presented is mean ± SEM. *$P<0.05$.

(D) Immunoprecipitation and western blot analysis for AMPK α1 phosphorylation at S485 and T172, and for AMPK β1/2 in hypothalamic lysates from WT and nestin-cre S6K1$^{fl/fl}$ (KO) mice treated with vehicle or i.p. 5 mg/kg leptin.

(E and F) Representative current-clamp traces from WT (E) and *Rps6kb1*-deleted (F) POMC neurons in the presence or absence of 50 nM leptin, where indicated. Expanded sections underneath are shown before and after the application of leptin.

(G-I) Representative current-clamp traces from WT (G) and *Rps6kb1*-deleted (H and I) AgRP neurons in the presence or absence of 50 nM insulin, where indicated. Expanded sections underneath are before and after the application of insulin.

(J) Bar charts showing the change in membrane potential (ΔVm) in WT (red bars) and KO (blue bars) POMC and AgRP neurons induced by leptin and insulin, respectively. Data is presented as mean ± SEM and is inclusive of non-responsive neurons. N=12-16 neurons per genotype. *$P<0.05$ from baseline recordings. No differences were observed between genotype.

(K and L) Representative traces from WT (K) and *Rps6kb1*-deleted (L) POMC neurons in the presence and absence of 5 mM leucine, where indicated.

Figure S4. Related to Figure 2, 3 & 4

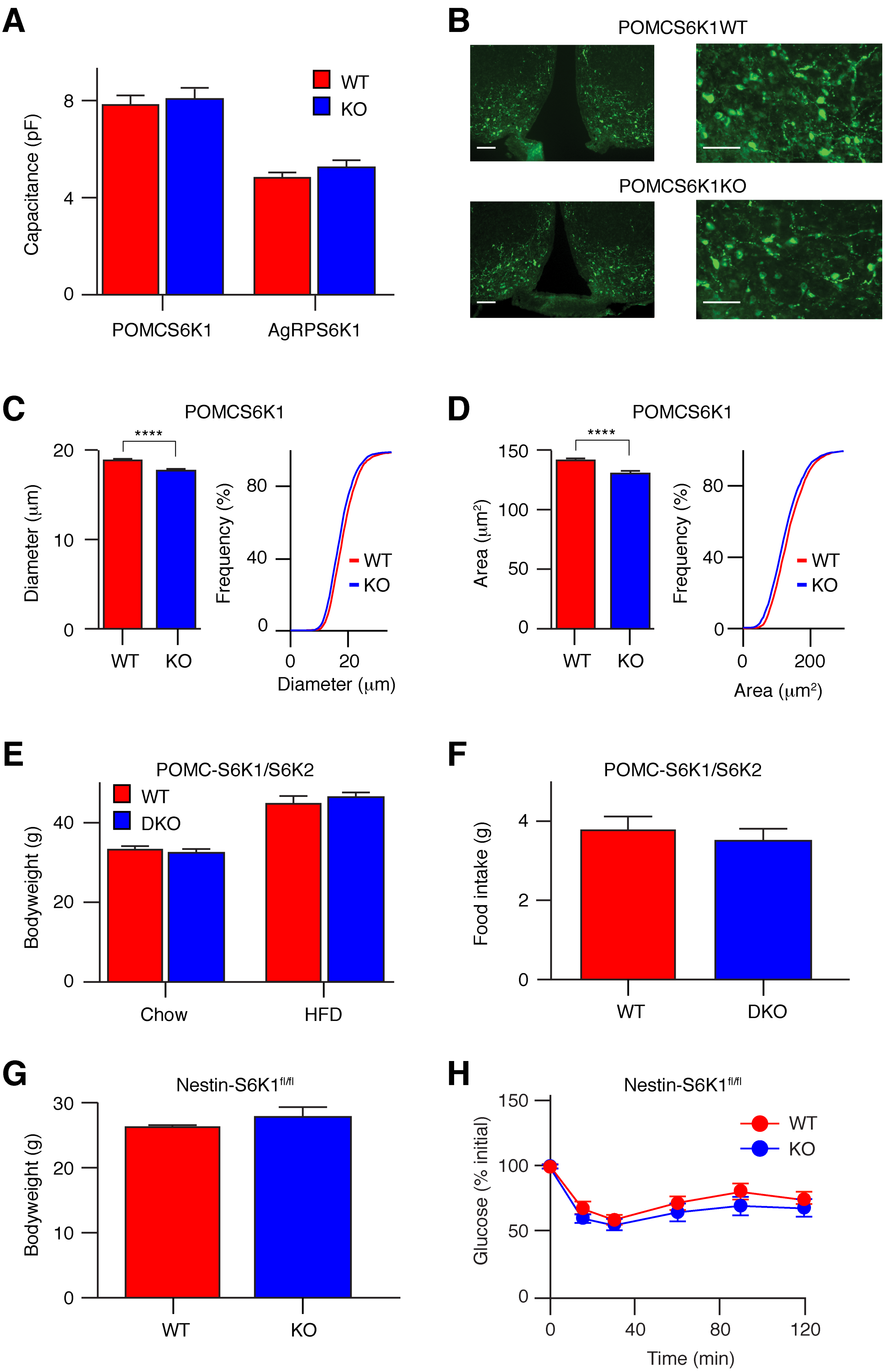

Figure S4. S6K1 in POMC neurons regulates cell body size but combined deletion of S6K1 and S6K2 in POMC neurons or deletion of S6K1 in all neurons does not alter bodyweight

(A) Whole-cell capacitance measurements from wild-type (WT, red bar) and knockout (KO, blue bar) POMCS6K1-GFP and AgRPS6K1-YFP neurons, N=22-38 neurons per genotype. Date presented is mean ± SEM.

(B) Representative low (left) and high (right) magnification images for POMC immuno-staining in WT (top) and *Rps6kb1*-deleted (bottom) POMC neurons. Scale bars represent 100 and 50 μm for low and high magnification images, respectively.

(C and D) Quantification of POMC soma diameter (C) and area (D) and calculated at equivalent coronal depths between WT and *Rps6kb1*-deleted cells. N=2471-3563 neurons from 4 mice per genotype. Data presented is mean ± SEM. ****P<0.0001.

(E) Bodyweight of male WT (red bar) and double KO (DKO, blue bar) POMC S6K1/S6K2 mice at 21 weeks on normal chow or high fat diet (HFD). N=17-24 mice per genotype. Data presented is mean ± SEM.

(F) Daily food intake in male WT (red bar) and DKO (blue bar) POMC S6K1/S6K2 mice on normal chow. N=6 mice per genotype. Data presented is mean ± SEM.

(G) Bodyweight of male WT (red bar) and KO (blue bar) nestin-cre $S6K1^{fl/fl}$ mice at 19 weeks on normal chow. N=13 mice per genotype. Data presented is mean ± SEM.

(H) Insulin-tolerance tests in WT (red circles) and KO (blue circles) male nestin $S6K1^{fl/fl}$ mice at 29 weeks of age on normal chow. N=12-13 mice per genotype. Data presented is mean ± SEM.

Table S1. CLAMS analysis of metabolism, energy expenditure and locomotion in wild-type (WT) and knockout (KO) POMCS6K1 and AgRPS6K1 mutant mice. Related to Figure 2

		POMCS6K1 Male		POMCS6K1 Female		AgRPS6K1 Male		AgRPS6K1 Female	
		WT (10)	KO (11)	WT (10)	KO (7)	WT (9)	KO (6)	WT (10)	KO (9)
VO_2 (ml/g/h)	Light	4.8±0.2	5.5±0.3	4.5±0.5	3.6±0.3	3.3±0.2	3.7±0.1	4.4±0.4	5.0±0.3
	Dark	5.6±0.3	5.6±0.3	5.2±0.6	4.0±0.4	3.9±0.3	4.4±0.2	4.7±0.4	5.2±0.3
VCO_2 (ml/g/h)	Light	4.8±0.2	5.0±0.3	4.2±0.5	3.2±0.3	3.0±0.2	3.4±0.1	4.1±0.4	4.7±0.3
	Dark	5.6±0.3	5.6±0.3	5.2±0.6	3.9±0.4	3.9±0.3	4.5±0.2	4.4±0.4	5.2±0.3
EE (Kcal/h)	Light	0.71±0.03	0.74±0.04	0.51±0.06	0.40±0.03	0.49±0.04	0.56±0.01	0.54±0.04	0.51±0.02
	Dark	0.85±0.05	0.82±0.05	0.60±0.08	0.46±0.04	0.59±0.05	0.68±0.02	0.57±0.05	0.54±0.03
RER (VCO_2/VO_2)	Light	0.87±0.01	0.91±0.02	0.91±0.02	0.92±0.01	0.90±0.01	0.92±0.01	0.91±0.01	0.92±0.01
	Dark	0.95±0.02	0.94±0.03	0.99±0.01	0.97±0.02	1.01±0.01	1.02±0.01	0.93±0.01	0.95±0.01
x-axis (count/min)	Light	16.2±2.4	22.6±3.5	21.3±2.8	19.1±2.4	17.6±2.8	12.8±2.6	28.7±3.2	29.8±3.1
	Dark	55.9±9.6	53.5±11.6	64.1±8.2	56.5±13.6	39.9±5.3	35.3±5.9	44.7±5.0	46.4±5.2
z-axis (count/min)	Light	1.1±0.3	1.2±0.2	2.3±0.7	2.4±0.4	1.2±0.2	1.0±0.2	9.7±3.9	11.4±2.9
	Dark	6.0±1.2	4.3±1.2	10.3±2.2	10.1±1.4	3.8±0.7	4.2±0.5	13.4±4.2	17.3±3.9

Data expressed as mean ± SEM during the light and dark phases. Number of mice per genotype shown in parenthesis. EE, energy expenditure; RER, respiratory exchange ratio.

Table S2. Blood glucose and serum insulin concentrations in wild-type (WT) and knockout (KO) POMCS6K1 and AgRPS6K1 mutant mice. Related to Figure 2

		POMCS6K1 Male		POMCS6K1 Female		AgRPS6K1 Male		AgRPS6K1 Female	
		WT	KO	WT	KO	WT	KO	WT	KO
Fed glucose (mM)	8 week old, Chow	8.8±0.3 (24)	9.0±0.3 (21)	8.7±0.3 (24)	8.7±0.2 (24)	10.5±0.3 (28)	10.2±0.3 (20)	9.3±0.2 (29)	9.6±0.3 (22)
	26 week old, Chow	9.2±0.3 (18)	9.6±0.2 (22)	7.9±0.3 (19)	8.2±0.3 (22)	11.0±0.3 (33)	10.3±0.3 (23)	10.4±0.4 (29)	9.5±0.3 (25)
	26 week old, HFD	11.4±0.5 (20)	10.7±0.3 (21)	8.5±0.2 (22)	8.6±0.2 (21)	12.9±0.5 (26)	12.7±0.5 (19)	9.9±0.3 (25)	9.7±0.3 (15)
Fasted glucose (mM)	8 week old, Chow	5.3±0.4 (15)	4.8±0.2 (22)	5.2±0.4 (17)	5.8±0.4 (19)	6.1±0.1 (30)	6.1±0.2 (23)	6.0±0.2 (27)	6.2±0.2 (18)
	26 week old, Chow	5.7±0.4 (18)	5.6±0.3 (22)	6.4±0.3 (19)	6.5±0.4 (22)	6.4±0.1 (31)	6.2±0.2 (23)	4.9±0.1 (31)	4.9±0.1 (25)
	26 week old, HFD	7.1±0.4 (20)	7.4±0.3 (21)	5.6±0.3 (22)	5.6±0.3 (21)	7.4±0.3 (25)	7.6±0.3 (19)	7.2 ±0.4 (24)	7.3±0.4 (15)
Fasted insulin (ng/ml)	Chow	0.53±0.11 (16)	0.79±0.17 (22)	0.31±0.05 (16)	0.38±0.05 (15)	0.38±0.03 (32)	0.49±0.11 (21)	0.58±0.08 (29)	0.70±0.23 (24)
	HFD	0.81±0.15 (19)	0.93±0.15 (21)	0.50±0.08 (15)	0.52±0.14 (17)	2.0±0.2 (26)	1.7±0.14 (19)	0.66±0.11 (24)	0.75±0.09 (13)

Data expressed as mean ± SEM. Number of mice per genotype shown in parenthesis. HFD, high fat diet

SUPPLEMENTAL EXPERIMENTAL PROCEDURES

Genotyping

For detection of Cre-mediated excision of exons 3 and 4 of *Rps6kb1* in the hypothalami of AgRPS6K1KO and POMCS6K1KO mice, genomic DNA was isolated from the hypothalamus, cortex, liver and skeletal muscle as previously described (Choudhury et al., 2005). The generation of a ~500 bp DNA product following PCR with primers TCCACCCACCAGTAAAGAGC and CCTCAGTCTCCTGAGTGTTAAGG is indicative of the floxed allele, whereas deletion is denoted by a ~450 bp band generated by primers TCCACCCACCAGTAAAGAGC and ACAAGAGGGCCAGTTGATGG.

Metabolic and food intake studies

Studies were performed in the animal's home cage unless indicated. Bodyweights from group-housed mice were measured weekly at 9-10am until 26 weeks of age. Glucose (i.p. 2 g/kg) and insulin (i.p. 0.75 U/kg) -tolerance tests were performed at 8 and 26 weeks of age, as previously described (Al-Qassab et al., 2009; Choudhury et al., 2005). At 34 weeks of age, fed mice were weighed prior to EchoMRI analysis of body composition. Fasted tail blood was analyzed for serum leptin (Millipore) and insulin (Crystal Chem.) by ELISA and for FFA (WAKO Chem.) and triglycerides (Sigma Aldrich). Serum corticosterone levels were determined by ELISA (Immunodiagnostics Systems) from fed mice. For food intake studies, mice were group housed until 10 weeks of age and then singly housed. Mice were allowed to acclimatize for 2 weeks and periodically fasted overnight to acclimatize them. Ad-libitum food intake was measured over 3 consecutive days and for 24 h following an overnight fast. Food intake was measured from singly housed mice injected with either vehicle or leptin (i.p. 0.3 mg/kg) at 9am and again at 6pm for 3 consecutive days. Food intake was also measured in fed mice for 8-24 hours following the injection of ghrelin (i.p. 5 mg/kg) or melanotan-II (MTII, i.p. 2 mg/kg) at 9am. Stainless steel cannulae (Plastics One Inc.) were stereotaxically implanted into the lateral cerebral ventricle so that the cannula tip was 0.4 mm posterior to bregma, 1.0 mm lateral to the midline and -2.1 mm ventral to the surface of the skull. Post-

surgery, the mice were singly housed, left for one week to recover and then habituated to overnight fasts for 2 weeks. Following an overnight fast, mice were injected with 0.5 μl of artificial cerebrospinal fluid (aCSF) or leucine (i.c.v. 2.2 μg) and food intake monitored over a 24 h period. All injections were performed with a 31-gauge stainless steel injector which projected 0.5 mm below the tip of the cannula. Following the infusion, the injector was left in place for an additional 30 s to allow the drugs to diffuse away from the cannula tip. Treatments, with either vehicle or drug, were crossed-over following a 1 week wash-out period. Correct cannula placement was confirmed at the end of the study by an increase in food intake after i.c.v. administration of NPY (1 μg). Mice were singly housed and habituated in CLAMS cages (Columbus Instruments) for 1-2 days prior to assessment of locomotion and energy expenditure over the subsequent 2 days.

Automated food intake monitoring

An episodic food intake monitoring apparatus (BioDAQ, Research Diets, Inc.) was used to assess feeding patterns in singly housed mice on normal chow. Food intake was measured and averaged from 5 consecutive days to obtain 24 h food intake kinetics. The BioDAQ system weighs the hopper with food (± 10 mg) every second and uses an algorithm to determine feeding bouts (changes in stable weight before and after a bout). Meals consist of one or more bouts separated by an inter-meal interval of 300 s with a minimum meal size of 20 mg.

Quantitative RT-PCR analysis

Tissues were lysed and homogenized in TRIzol reagent (Ambion) and total RNA was isolated using the RNeasy mini kit (Qiagen). First-strand cDNA was generated using Taqman reverse transcription reagents (Applied Biosystems) and qPCR was performed using Taqman universal PCR mastermix in a 7900HT real-time PCR system (Applied Biosystems). mRNA quantities were normalized to *Hprt*, *Ppia* or *Gapdh* after determination by the comparative Ct method. Primers used were: *Abcc8* (Mm00803450), *Agrp*

(Mm00475829_g1), *Cpe* (Mm00516341_m1) *Dgat2* (Mm00499536_m1), *Dio2* (Mm00515664_m1), Elov3 (Mm00468164_m1) *Fabp4* (Mm00445878_m1), *Fasn* (Mm00662319_m1), *Gabra1* (Mm00439046_m1), *Gabra2* (Mm00433435_m1), *Gabra3* (Mm01294271_m1), *Gabra5* (Mm00621092_m1), *Gapdh* (Mm99999915_g1), *Gck* (Mm00439129_m1), *Hprt* (Mm00446968_m1), *Lipe* (Mm00495359_m1), *IL6* (Mm00446190-m1), *Kcnj11* (Mm00440050_m1), *Mc4r* (Mm00457483_s1), *Npy* (Mm00445771_m1), *Pck1* (Mm01247058_m1), *Pgc1a* (Mm00447183_m1), *Plin1* (Mm00558672_m1), *Pomc* (Mm00435874_m1), *Ppia* (Mm03302254_g1), *Scd1* (Mm00772290_m1), *Ucp1* (Mm01244861_m1).

Hypothalamic immunohistochemistry

Immunohistochemistry was performed as previously described (Al-Qassab et al., 2009; Choudhury et al., 2005). Fasted mice were injected with leptin (i.p. 5 mg/kg) and perfused with paraformaldehyde (4% w/v). Arcuate sections were incubated with rabbit anti-POMC precursor (1:1000; Phoenix Pharmaceuticals Inc.) and detection performed using a secondary antibody coupled to Alexa-Fluor-488. After extensive washing, slices were incubated with a rabbit anti-pSTAT3 (Tyr705) antibody (1.1000, Cell Signaling) which was detected using an ABC detection kit (Vector labs). Fluorescent images were taken with an epifluorescence microscope fitted with a color digital camera. Mice expressing GFP (POMC-GFP mouse) or YFP (AgRPCre-YFP) were fixed and arcuate sections incubated with a rabbit p70 S6 kinase (49D7, Cell Signaling) primary antibody (1:200) followed by a secondary antibody coupled to Alexa-Fluor-594. POMC neuronal measurements were counted from labeled cells using ImageJ software.

Electrophysiology

Hypothalamic coronal slices (350 µm) were cut from aged (3 or 7 month old) matched transgenic mice expressing POMCCre/POMC-GFP or AgRPCre/Rosa26YFP with or without floxed *Rps6bk1*. Slices were maintained at room temperature (22-25ºC) in an external

solution containing (in mM) NaCl 125, KCl 2.5, NaH_2PO_4 1.25, $NaHCO_3$ 25, $CaCl_2$ 2, $MgCl_2$ 1, D-glucose 10, D-mannitol 15, equilibrated with 95% O_2, 5% CO_2, pH 7.4. POMC and AgRP neurons were visualized in the arcuate nucleus by the expression and excitation of GFP and YFP, respectively. Whole-cell current-clamp (I_{fast}) recordings were made at ~35°C using borosilicate glass pipettes (4-8 MΩ) containing (in mM) Kgluconate 130, KCl 10, EGTA 0.5, NaCl 1, $CaCl_2$ 0.28, $MgCl_2$ 3, Na_2ATP 3, GTP 0.3, phosphocreatine 14 and HEPES 10 (pH 7.2), as previously described (Al-Qassab et al., 2009; Choudhury et al., 2005). Following a minimum of 10 min of stable recording, hormones were applied for 2-3 min using a broken tipped pipette (~3 μm) positioned above the recording neuron. Stock reagents were diluted (≥1000 fold) in a modified external solution with $NaHCO_3$ replaced with HEPES (10 mM, pH 7.4). Stocks of recombinant leptin (R&D Systems) and insulin (Novo-Nordisk Inc.) were diluted in HEPES-buffered external solution. All other reagents were purchased from Sigma-Aldrich. External glucose was replaced with mannitol to maintain osmolarity and biophysical properties were obtained from separate recordings in different glucose concentrations. Neurons were voltage-clamped at -70 mV using a modified internal solution in which Kgluconate was replaced with CsCl (130 mM). Slices were bathed in tetrodotoxin (1 μM) and (+)-bicuculline (20 μM) for miniature excitatory currents (mEPSC) or NBQX (5 μM, 2,3-dihydroxy-6-nitro-7-sulfamoyl-benzo[f]quinoxaline-2,3-dione) and AP5 (50 μM (2*R*)-amino-5-phosphonovaleric acid; (2*R*)-amino-5-phosphonopentanoate) for miniature inhibitory currents (mIPSC). Note that that there were no statistical differences in the excitable properties of POMC neurons in young and old mice (Vm; 3 month, WT: -48.6±1.3 vs KO: -53.3±1.6 mV, n=24-29; 7 month, WT: -49.6±2.0 vs KO: -54.1±1.3 mV, n=9-16).

Hyperinsulinemic-euglycemic clamps studies

Clamps were conducted as previously described (Voshol et al., 2001). Animals were anesthetized by intraperitoneal injection of a combination of 6.25 mg/kg acetylpromazine, 6.25 mg/kg midazolam and 0.31 mg/kg fentanyl. An infusion needle was placed into the tail

vein and D-[^{3}H] glucose (specific activity: 10-20Ci (370-740GBq)/mmol) was infused at a rate of 0.006 MBq/min for 60 min to achieve steady-state levels. Thereafter, insulin (Actrapid; Novo Nordisk) was infused at a constant rate of 0.09 mU/min after a bolus dose of 3.3 mU and D-[^{3}H]-glucose was continued at a rate of 0.006 MBq/min. A variable infusion of 12.5% D-glucose was used to maintain blood glucose at euglycemic (basal) levels. Blood glucose was measured with an AlphaTRAK glucometer (Abbott Animal Health) every 5-10 minutes and glucose infusion adjusted accordingly. After 50 minutes from the start of the insulin infusion, ^{14}C-2-Deoxy-glucose-phosphate (Specific Activity: 250-350mCi (9.25-13.0GBq)/mmol) was administered i.v. to assess tissue-specific glucose uptake. Steady-state was reached after 90 minutes and blood samples were taken at 10 minutes intervals over 30 minutes to determine steady-state levels of [^{3}H]-glucose. Mice were then killed by cervical dislocation and the organs removed and frozen. Haematocrit was measured at baseline and after the clamp (no significant changes were observed between each genotype and their littermate controls, data not shown). To measure plasma [^{3}H]-glucose, proteins were precipitated with trichloroacetic acid (final concentration 10%), centrifuged, and supernatant dried and re-suspended in water. The samples were counted using scintillation counting (Hidex Scintilation counter, LabLogic). Tissue samples were homogenized (~5-10% wet wt/vol, depending on tissue) in 0.5% percholic acid, centrifuged, supernatants neutralized, and ^{14}C-2-Deoxy-glucose-phosphate precipitated using BaOH/$ZnSO_4$. Total and precipitated counts of supernatants were subtracted and plasma ^{14}C-2-Deoxy-glucose-phosphate counts were used to calculate tissue specific uptake. Protein content in homogenates was performed using DC protein assay (BioRad). The glucose turnover rate (μmol/min) was calculated during the basal period and under steady-state clamp conditions as the rate of tracer infusion (dpm/min) divided by the plasma specific activity of [^{3}H] glucose (dpm/μmol). The hyperinsulinemic hepatic glucose production was calculated as the difference between the tracer-derived rate of glucose appearance and the glucose infusion rate.

Western blot analysis

Tissues were removed and homogenized in lysis buffer (50 mM Tris pH 7.4, 150 mM NaCl, 1 mM EDTA, 1% w/v Triton X-100) supplemented with Roche complete protease inhibitor cocktail and phosphatase inhibitors (1 mM sodium orthovadanate, 5 mM sodium fluoride and 2 mM β-glycerophosphate). 20-100 μg of total protein homogenates were run on 15% SDS-PAGE gels, transferred to PVDF membranes and blotted with antibodies against p70 S6 Kinase (1:3000, #2708 Cell Signaling), total S6 Ribosomal Protein (1:1000, #2217 Cell Signaling), phospo-S6 Ribosomal Protein Ser240/244 (1:1000, #2708 Cell Signaling), and tubulin as a loading control (1:10,000, #T5293 Sigma). Detection was performed using enhanced chemiluminescence (Luminata Crescento, Millipore) and exposed on films. The intensity of the bands was quantified by densitometry using ImageJ software and normalized to either β-tubulin or total S6 ribosomal protein. For AMPK studies, hypothalamic tissue was homogenized in buffer (50 mM Tris-HCl pH 8.4 (at 4°C), 50 mM NaF, 5 mM NaPP, 0.25 M sucrose (or mannitol), 1 mM EDTA, 1 mM benzamidine, 0.1 mM PMSF, 1 mM DTT) and supernatants were either western blotted or used for immunoprecipitation (500 μg). Lysates were pre-cleared using protein G sepharose and then immunoprecipitated using a sheep antibody specific for AMPK α1. The immune-complexes were washed and run on SDS PAGE gels (Novex precast Tris/Bis gels). Antibodies used for blotting AMPKβ1/2, AMPKp-T172 and AMPKpS485/491 were all from Cell Signaling.

General experimental approaches

Where possible, investigators were blinded to the genotype of both study animals and that of tissue and blood samples. For experiments involving treatments, mice were randomized by genotype to study groups or a cross-over design was used where indicated and study cohorts were matched for initial bodyweight where appropriate. Treatments were administered in random order. All metabolic studies were replicated in at least 2 independent cohorts. Study cohort sizes were determined by power calculations based on our previous data in mice with targeted hypothalamic mutations. In electrophysiological experiments, a

typical mouse allowed 1-5 recordings but observations were repeated on at least 4 different mice.

SUPPLEMENTAL REFERENCES

Al-Qassab, H., Smith, M.A., Irvine, E.E., Guillermet-Guibert, J., Claret, M., Choudhury, A.I., Selman, C., Piipari, K., Clements, M., Lingard, S., *et al.* (2009). Dominant role of the p110beta isoform of PI3K over p110alpha in energy homeostasis regulation by POMC and AgRP neurons. Cell Metab *10*, 343-354.

Choudhury, A.I., Heffron, H., Smith, M.A., Al-Qassab, H., Xu, A.W., Selman, C., Simmgen, M., Clements, M., Claret, M., Maccoll, G., *et al.* (2005). The role of insulin receptor substrate 2 in hypothalamic and beta cell function. J Clin Invest *115*, 940-950.

Claret, M., Smith, M.A., Knauf, C., Al-Qassab, H., Woods, A., Heslegrave, A., Piipari, K., Emmanuel, J.J., Colom, A., Valet, P., *et al.* (2011). Deletion of Lkb1 in pro-opiomelanocortin neurons impairs peripheral glucose homeostasis in mice. Diabetes *60*, 735-745.

Voshol, P.J., Jong, M.C., Dahlmans, V.E., Kratky, D., Levak-Frank, S., Zechner, R., Romijn, J.A., and Havekes, L.M. (2001). In muscle-specific lipoprotein lipase-overexpressing mice, muscle triglyceride content is increased without inhibition of insulin-stimulated whole-body and muscle-specific glucose uptake. Diabetes *50*, 2585-2590.
